# β-Catenin transcriptional activity is required for establishment of inner pillar cell identity during cochlear development

**DOI:** 10.1371/journal.pgen.1010925

**Published:** 2023-08-28

**Authors:** Michael Ebeid, Ippei Kishimoto, Pooja Roy, Mohd Ali Abbas Zaidi, Alan G. Cheng, Sung-Ho Huh

**Affiliations:** 1 Holland Regenerative Medicine Program, University of Nebraska Medical Center, Omaha, Nebraska, United States of America; 2 Department of Neurological Sciences, University of Nebraska Medical Center, Omaha, Nebraska, United States of America; 3 Department of Otolaryngology-Head and Neck Surgery, Stanford University School of Medicine, Stanford, California, United States of America; 4 Department of Otolaryngology-Head and Neck Surgery, University of Mississippi Medical Center, Jackson, Mississippi, United States of America; NIH/NIDCD, UNITED STATES

## Abstract

The mammalian cochlea is composed of sensory hair cells as well as multiple different types of non-sensory supporting cells. Pillar cells are one type of supporting cell that form the tunnel of Corti and include two morphologically and functionally distinct subtypes: inner pillar cells (IPCs) and outer pillar cells (OPCs). The processes of specification and differentiation of inner versus outer pillar cells are still unclear. Here, we show that β-Catenin is required for establishing IPC identity in the mammalian cochlea. To differentiate the transcriptional and adhesion roles of β-Catenin in establishing IPC identity, we examined two different models of *β-Catenin* deletion; one that deletes both transcriptional and structural functions and one which retains cell adhesion function but lacks transcriptional function. Here, we show that cochleae lacking β-Catenin transcriptional function lost IPCs and displayed extranumerary OPCs, indicating its requirement for establishing IPC identity. Overexpression of β-Catenin induced proliferation within IPCs but not ectopic IPCs. Single-cell transcriptomes of supporting cells lacking β-Catenin transcriptional function show a loss of the IPC and gain of OPC signatures. Finally, targeted deletion of β-Catenin in IPCs also led to the loss of IPC identity, indicating a cell autonomous role of β-Catenin in establishing IPC identity. As IPCs have the capacity to regenerate sensory hair cells in the postnatal cochlea, our results will aid in future IPC-based hair cell regeneration strategies.

## Author summary

The organ of Corti, the hearing apparatus, requires a precise arrangement of various cell types including hair cells (required for sound reception) and supporting cells (supporting hair cell function) for proper function. β-Catenin is an essential component of the Wnt/β-Catenin signaling pathway, which is required for normal development of different cell types. Here, we show that β-Catenin is required for differentiation of the supporting cell subtype inner pillar cells. Loss of transcriptional activity of β-Catenin caused loss of inner pillar cells and gain of outer pillar cell-like cells. In addition, we show that β-Catenin works in a cell autonomous manner within pillar cells to establish inner pillar cell identity. These findings advance our understanding of the mechanisms involved in cell identity establishment during cochlear development and thereby will guide regenerative efforts to repair damaged cells within the organ of Corti.

## Introduction

**Fig 1:**
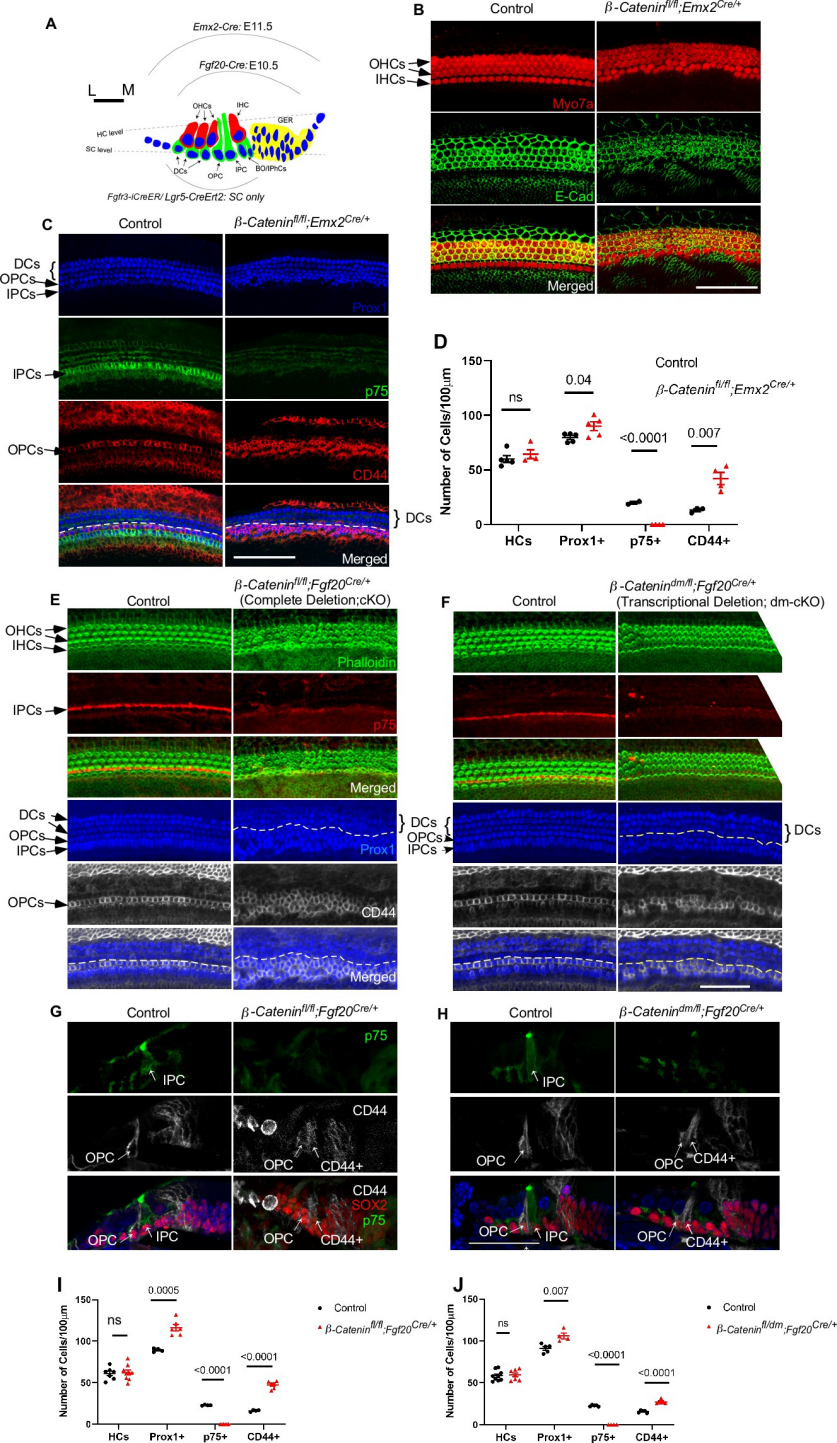
β-Catenin regulates supporting cell development. (A) Diagram showing different cell types within the organ of Corti, including inner hair cells (IHCs), outer hair cells (OHCs), inner border cells (Bo), inner phalangeal cells (IPh), inner pillar cells (IPC), outer pillar cells (OPC), and Deiters’ cells (DC). Dashed lines show the plane of imaging for hair cells and supporting cells. Arcs showing the activity domain of different Cre drivers (B) Immunostaining of whole mount E18.5 cochlear epithelium (basal turn) showing hair cells (Myo7a: red) and E-cadherin (green) in *β-Catenin* cKO (*β-Catenin*^*fl/fl*^*; Emx2*^*Cre/+*^) versus control mice, showing disorganized hair cells and disrupted medial-lateral boundaries in *β-Catenin* cKO. (C) Immunostaining for supporting cell marker Prox1 (blue), IPC marker p75 (green) and OPC marker CD44 (red) in *β-Catenin* cKO versus control showing loss of p75 staining and an increased number of CD44+ cells in *β-Catenin* cKO. White dashed lines indicate the boundary between the first row of Deiters’ cells and the OPCs (D) Quantification of HCs, Prox1+, p75+, and CD44+ cells at E18.5 in *β-Catenin* cKO versus control (apical, middle, and basal turns averaged). (E-F) Immunostaining of whole mount E18.5 cochlear epithelium (basal turn) from *β-Catenin*^*fl/fl*^*; Fgf20*^*Cre/+*^ (Complete deletion; cKO) and *β-Catenin*^*dm/fl*^*; Fgf20*^*Cre/+*^ (Transcriptional deletion; dm-cKO) for phalloidin, p75, Prox1 and CD44 showing loss of p75 staining, increased CD44 staining and increased density of Prox1+ cells in both deletion models versus controls. (G-H) Immunostaining of cochlear sections for p75 (green), CD44 (gray), and Sox2 (red) at E18.5 from both deletion models versus control showing loss of the IPC maker and gain of the OPC marker. White dashed lines indicate the boundary between the 1^st^ row of Deiters’ cells and the OPCs (I-J) Quantification of the density of HCs, Prox1+, p75+, and CD44+ cells at E18.5 in both deletion models versus controls showing a statistically significant loss of p75+ cells and gain of CD44+ cells. Scale bar = 100μm. n = 3–5 per group, mean±SE, Student’s t-test p-value indicated.

The sensory epithelium of the mammalian cochlea (the organ of Corti) consists of multiple unique cell types arranged in a highly ordered pattern that is necessary for proper auditory function. Cells in this organ are categorized into sensory hair cells and non-sensory supporting cells. Hair cells are arranged into 4 rows: three rows of outer hair cells and one row of inner hair cells. Supporting cells consist of multiple cell types including (from lateral to medial): several rows of Hensen’s cells, 3 rows of Deiters’ cells (DCs) that underlie the outer hair cells, one row of outer pillar cells (OPCs), one row of inner pillar cells (IPCs) and few rows of inner phalangeal cells that support inner hair cells (reviewed in [1] and displayed in Fig 1A). In the mature organ, IPCs and OPCs form the boundaries of a triangular space called the tunnel of Corti [2].

Supporting cells play crucial roles in cochlear development, maturation, and repair after injury. During development, supporting cells regulate patterning of the epithelium and synaptogenesis [3–6]. Supporting cells provides structural support during sound stimulation, maintain extracellular ion concentrations essential for hair cell function, maintain the epithelial reticular lamina, and support the survival of hair cells and neurons [7–10]. After damage to the auditory epithelium, supporting cells regulate scar formation and clearance of debris [11]. Additionally, it has been shown that supporting cells can replace hair cells after damage in the neonatal mouse cochlea, yet this ability decreases significantly during the postnatal period [12–14].

During mammalian cochlear development, both hair cells and supporting cells are derived from the prosensory cells that are specified in a narrow domain in the floor of the cochlear duct [15]. Around embryonic day (E)13, prosensory cells begin to exit mitosis and upregulate the cell cycle inhibitor CDKN1B (p27) [16]. Once hair cells are specified, Notch signaling mediates lateral inhibitory interactions to prevent prosensory cells from adopting a hair cell fate, which creates the mosaic hair cell/supporting cell pattern in the mature cochlea [17–19]. As hair cells differentiate, surrounding supporting cells differentiate [20,21]. There is emerging evidence that distinct mechanisms regulate specification of different subtypes of supporting cells. For instance, multiple studies have shown that pillar cell fate is induced by the FGF8 secreted from inner hair cells around E16.5. FGF8 binds to and activates FGFR3 in adjacent precursors of supporting cells, leading to the formation of pillar cells [22–27]. However, how inner versus outer pillar cells are individually specified is still unknown.

Canonical Wnt/β-Catenin signaling regulates a wide range of developmental processes including cell proliferation, migration, epithelial-mesenchymal transition, and differentiation [28,29]. In the absence of Wnt, cytoplasmic β-Catenin protein is constantly degraded by the action of the Axin complex, which prevents β-Catenin from reaching the nucleus. Once a Wnt ligand binds to a Frizzled (Fz) receptor and its co-receptor, a complex is formed that eventually leads to the inhibition of Axin-mediated β-Catenin degradation and to the stabilization of β-Catenin, which in turn accumulates and translocates to the nucleus to form complexes with transcription factors such as Tcf and Lef and thereby activates Wnt target gene expression (reviewed in [28,30]). In addition to its role in canonical Wnt signaling, β-Catenin connects E-cadherin and α-Catenin to generate an important component in the cell-to-cell adhesion system which is required for the structural integrity of epithelial cells [31].

There is growing evidence that Wnt/β-Catenin signaling pathway is essential for mammalian cochlear development. In the developing cochlea, canonical Wnt/β-Catenin signaling reporter activity becomes restricted to medial supporting cells including pillar cells and inner phalangeal cells [32]. In two independent studies, deletion of *β-Catenin* during differentiation of prosensory cells (E13.5) resulted in loss of NGFR (p75) expression in the IPCs [33,34]. Additionally, it is shown that *β-Catenin* deletion, via its cell adhesion role, disrupts the boundaries between medial and lateral compartments of the developing cochlea [34]. To differentiate the canonical Wnt signaling from the structural roles of β-Catenin in supporting cell development, we have examined two different *β-Catenin* deletion alleles in this study: (1) the *β-Catenin*^*fl*^ allele that has a deletion from exon 2 (which contains the ATG translation start site) to exon 6, generating a *β-Catenin* null allele that deletes both transcriptional and structural functions [35], and (2) the *β-Catenin*^*dm*^ allele which retains cell adhesion function but deletes transcriptional function by point mutating Aspartic Acid of 164 to Alanine (D164A) and deleting the C-terminus [36]. In this study, we analyzed the differences between these two *β-Catenin* deletion models, to determine the role of β-Catenin in pillar cell fate determination. Through genetic modification of *β-Catenin* gene, lineage tracing, and single cell RNA sequencing, we showed that that β-Catenin transcriptional activity within supporting cells is required to establish IPC identity during cochlear development.

## Results

### β-Catenin transcriptional activity regulates pillar cell development

In our previous study [34], we utilized *β-Catenin*^*fl*^ allele to eliminate both the transcriptional and cell adhesion functions of β-Catenin, and showed that *Sox2*-Cre- and *Fgf20*-Cre-mediated deletions of *β-Catenin* caused hair cell and supporting cell disorganization throughout the cochlear duct. Additionally, p75 expression (IPC marker) was downregulated or absent. To further investigate the role of β-Catenin in the development of cochlear supporting cells, we utilized an additional Cre driver, *Emx2-*Cre, which is active in cochlear epithelial cells by E12.5. *Emx2*-Cre has a broader activity domain and higher efficiency compared to the *Sox2-Cre* and *Fgf20*-Cre models, as well as higher animal survival rates [34,37,38]. Analysis of cochleae from β-Catenin conditional mutants (*β-Catenin*^*fl/fl*^*; Emx2*^*Cre/+*^) and littermate controls (*β-Catenin*^*fl/+*^*; Emx2*^*Cre/+*^, *β-Catenin*^*fl/+*^*; Emx2*^*+/+*^, or *β-Catenin*^*fl/fl*^*; Emx2*^*+/+*^) at E18.5 showed hair cell disorganization in all cochlear turns, disrupted boundaries between inner and outer hair cells (as indicated by E-cadherin staining), without a significant change in hair cell density (Fig 1A and 1B), a phenotype consistent with our previous results [34]. On the other hand, *β-Catenin* mutants revealed an increase in Prox1+ supporting cell density, loss of IPCs (p75+ cells), and gain of OPCs (CD44+ cells) across all cochlear turns (n = 4, Student’s t-test p<0.05) (Fig 1C and 1D). Most ectopic CD44+ cells were located medial to the first row of Prox1+ Deiters’ cells (Fig 1C). To investigate the underlying cause of increased Prox1+ cell density, we immunostained for Ki67 proliferation marker and Sox2, and found no significant changes in Sox2/Ki67 double positive cells in the prosensory domain in *β-Catenin* conditional mutants. This indicates that the increase in Prox1+ cell density is not due to increased cell proliferation (S1A and S1B Fig). Altogether, our data suggest that β-Catenin is required for IPC identity within the developing cochlea.

To test whether the phenotype observed in *β-Catenin* conditional mutant cochleae is due to the deletion of *β-Catenin* cell adhesion or transcriptional function, we generated and compared 2 different mouse models: *β-Catenin*^*fl/fl*^*; Fgf20*^*Cre/+*^ (complete deletion; cKO) to delete both *β-Catenin* cell adhesion and transcriptional functionalities, and *β-Catenin*^*dm/fl*^*; Fgf20*^*Cre/+*^ (transcriptional deletion; dm-cKO) to delete *β-Catenin* transcriptional activity while retaining the cell-cell adhesion function [36]. Since homozygous *β-Catenin*^*dm/dm*^ mice exhibit early embryonic lethality [36], we utilized *Fgf20*^*Cre*^ mice to delete one copy of *β-Catenin*^*fl*^ allele, while the other allele is *β-Catenin*^*dm*^ (*β-Catenin*^*dm/fl*^*; Fgf20*^*Cre/+*^). *β-Catenin*^*fl/+*^*; Fgf20*^*Cre/+*^ was used as the control for both cKO and dm-cKO models. Both models have demonstrated high efficiency of β-Catenin deletion from the cochlear supporting cells [34]. Postnatal day (P)0 cochleae from both models (cKO and dm-cKO) showed loss of p75+ IPCs and gain of CD44+ OPCs along with an overall increase in density of Prox1+ supporting cells (Fig 1E–1J) (n = 5, Student’s t-test p<0.05). Hair cells and supporting cells appeared less disorganized in the dm-cKO model compared to the cKO model, likely because the dm-cKO cochleae retain the cell adhesion function of β-Catenin. Immunostaining of cochlear sections from both models confirmed that supporting cells in the region of IPCs have lost expression of p75 and gained CD44. Of note, we observed fewer supernumerary CD44+ cells in dm-cKO compared to cKO cochleae (Fig 1I and 1J), which may indicate a less severe phenotype in the former model. It is notable that these supporting cells retained Sox2 expression, indicating that they maintained their supporting cell signature (Fig 1G and 1H). Since both cKO and dm-cKO models displayed the loss of IPC staining and gain of OPC staining, β-Catenin transcriptional activity is likely required for establishing IPC fate.

### Lineage tracing of *β-Catenin*-deficient cells shows that β-Catenin transcriptional activity is required for IPC fate

**Fig 2:**
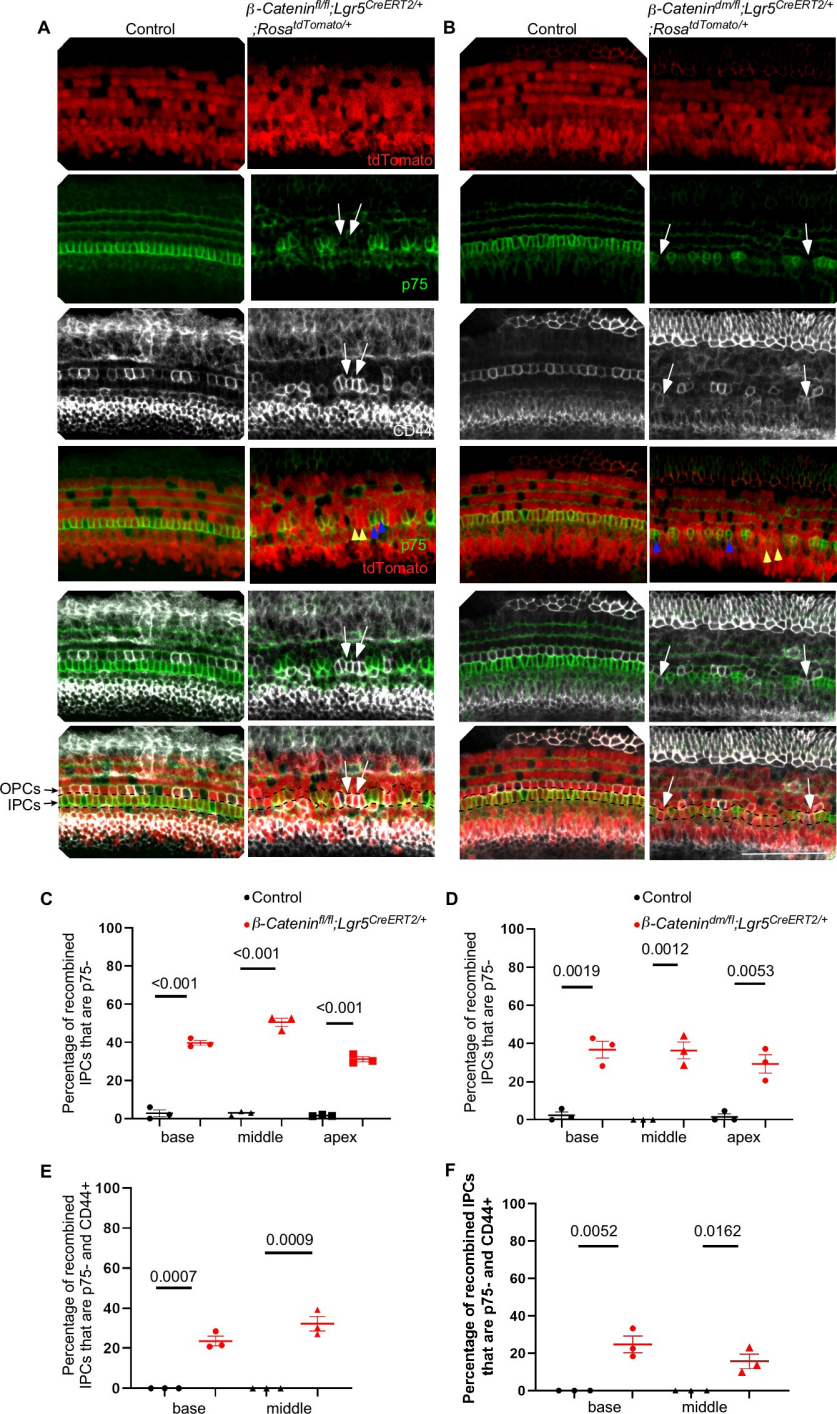
β-Catenin transcriptional activity is required for inner pillar cell identity. (A) Immunostaining of whole mount E18.5 cochlear epithelium from *β-Catenin*^*fl/fl*^*; Lgr5*^*CreERT2/+*^*; Rosa*^*tdTomato/+*^ cochlea compared to control (*β-Catenin*^*fl/+*^*; Lgr5*^*CreERT2/+*^*; Rosa*^*tdTomato/+*^) induced at E14.5 showing basal turn stained for p75 (green) and CD44 (gray) along with tdTomato fluorescence (red) in cells with *Lgr5*^*CreERT2*^ recombination. Red/green merged images show recombined IPCs that lost p75 staining (yellow arrowheads), and non-recombined cells maintaining p75 staining (blue arrowheads). White arrows point to a subset of recombined cells within the IPC region (region between the black dashed lines) lacking p75 staining and expressing CD44 in *β-Catenin*^*fl/fl*^*; Lgr5*^*CreERT2/+*^*; Rosa*^*tdTomato/+*^ cochlea. (B) Immunostaining of whole mount E18.5 cochlear epithelium (basal turn) from *β-Catenin*^*fl/dm*^*; Lgr5*^*CreERT2/+*^*; Rosa*^*tdTomato/+*^ cochlea compared to control showing similar results as (A). (C-D) Quantification of the percentage of recombined IPCs that are p75- in each turn from both models. (E-F) Quantification of the percentage of recombined IPCs that are p75- and CD44+ in each turn from both models. Scale bar = 50μm. n = 3, mean±SE, Student’s t-Test p-value indicated.

To concurrently trace and delete *β-Catenin* in prosensory cells around the time of pillar cell specification, we utilized an inducible Cre allele (*Lgr5*^*CreERT2/+*^) that is active in the Sox2+ supporting cells of the cochlear duct [39], along with *Rosa*^*tdTomato/+*^ allele that expresses tdTomato upon Cre recombinase activity to trace Cre activity [40]. This setup allows spatial and temporal control for *β-Catenin* deletion at later time points compared to Fgf20-Cre line. We generated pregnant female mice carrying *β-Catenin*^*fl/fl*^*; Lgr5*^*CreERT2/+*^*; Rosa*^*tdTomato/+*^ embryos (lacking both *β-Catenin* functions) and *β-Catenin*^*fl/dm*^*; Lgr5*^*CreERT2/+*^*; Rosa*^*tdTomato/+*^ embryos (lacking *β-Catenin* transcription but retaining cell adhesion function). Pregnant females were induced with tamoxifen (250 mg/kg) once at E14.5, before pillar cell differentiation. Analysis of the cochleae from control mice at E18.5 for the recombination efficiency within the region of IPCs showed efficient recombination ranging from 75% to 82% in both models (Fig 2A and 2B). We further validated *β-Catenin* deletion using cochlear immunostaining against β*-*Catenin 48 hours post induction (E16.5) (S2A and S2B Fig). The majority of IPCs along with other supporting cells showed loss of β-Catenin staining in the complete deletion model (*β-Catenin*^*fl/fl*^*; Lgr5*^*CreERT2/+*^*; Rosa*^*tdTomato/+*^). On the other hand, β-Catenin staining was present in the transcription deletion model (*β-Catenin*^*fl/dm*^*; Lgr5*^*CreERT2/+*^*; Rosa*^*tdTomato/+*^), as β-Catenin cell adhesion function is expected to be preserved in this model. Analysis of *β-Catenin* deficient cochlea showed loss of p75 staining from many recombined (tdTomato+) cells within the region of IPCs in both conditional deletion models when compared to controls (Fig 2A and 2B). In complete deletion cochleae, the average percentages of recombined IPCs that lost the p75 staining were significantly higher (39.7% (base), 50.6% (middle), and 31.2% (apex)) than those in controls (2.6% (base), 2.7% (middle), and 1.6 (apex), Fig 2C) (n = 3, mean, Student’s t-test p<0.05). Similarly, in transcription deletion cochleae, the average percentages of recombined IPCs that lost the p75 staining were significantly higher (36.7% (base), 36.3% (middle), and 29.3% (apex)) compared to controls (2.3% (base), 0% (middle), and 1.6% (apex), Fig 2D) (n = 3, mean, Student’s t-test p<0.05). Of note, we observed that some recombined cells in IPC region maintained p75 staining, which is likely because not all recombined cells have deleted β-Catenin. This is supported by the fact that some recombined cells maintained β-Catenin expression (S2A and S2B Fig). Nevertheless, these results affirm that β-Catenin transcriptional activity is required for establishing IPC fate. Next, to assess IPC to OPC conversion, we analyzed the percentage of recombined IPCs that lost p75 and gained CD44 expression. Because CD44 expression has not yet matured in the apical turn of E18.5 cochlea, we analyzed the basal and middle turns only. In complete deletion cochleae, 23.6% (basal turn) and 32.3% (middle turn) of recombined IPCs lost p75 and gained CD44 staining, whereas none was observed in controls. Similarly, transcription deletion cochleae, some recombined IPCs lost p75 and gained CD44 staining (24.7% (basal turn) and 15.6% (middle turn)) whereas none was detected in controls (n = 3/group, mean, Student’s t-test p<0.05) (Fig 2E and 2F). These results affirm that β-Catenin transcriptional activity is required for establishing IPC fate. Furthermore, these results indicate that a subset of β-Catenin deficient cells that failed to differentiate to IPCs had likely acquired an OPC fate.

### β-Catenin overexpression induces IPC proliferation but not ectopic IPC formation

**Fig 3:**
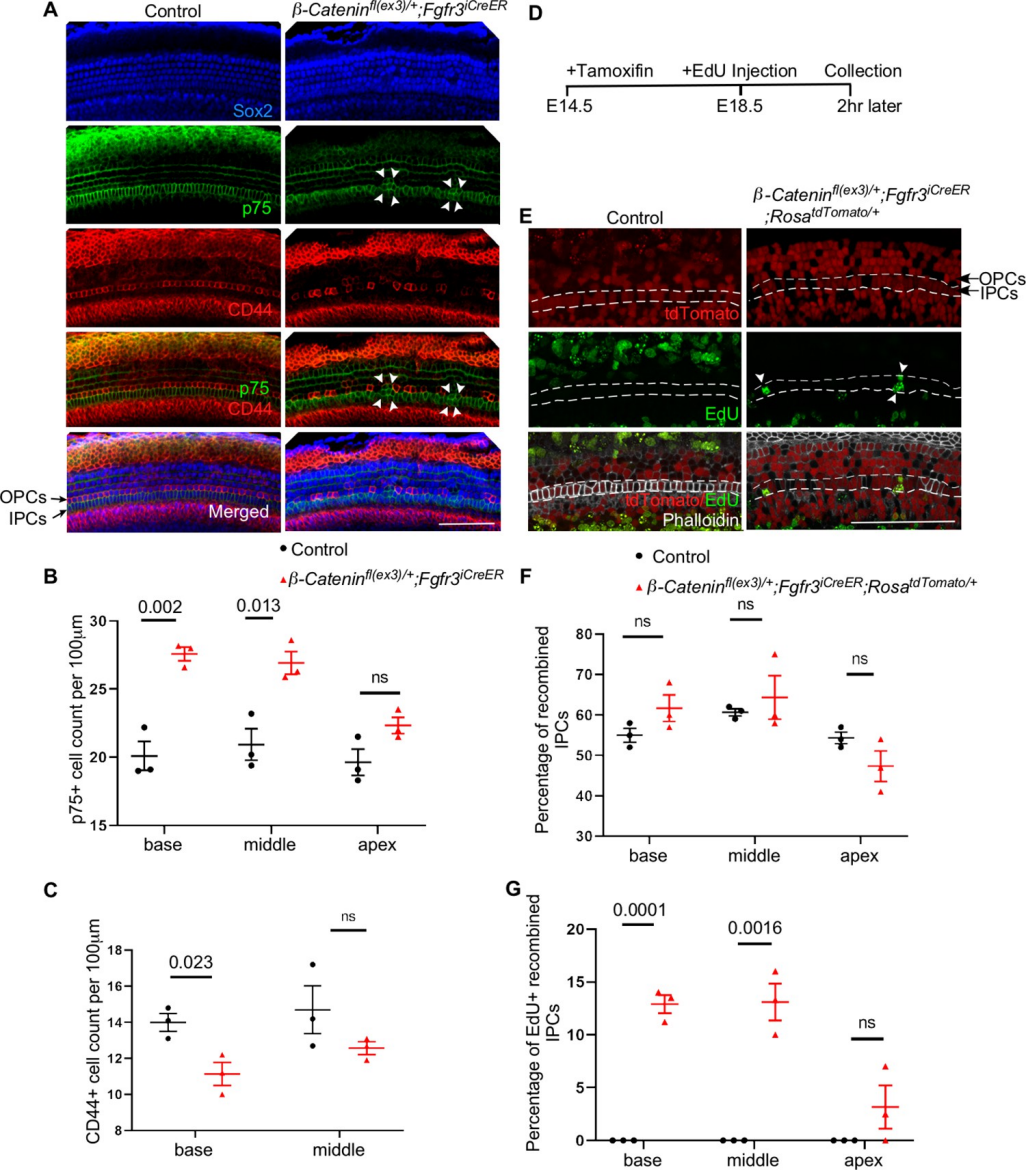
Constitutively active β-Catenin within supporting cells induce inner pillar cell proliferation. (A) Immunostaining of whole mount E18.5 cochlear epithelium from constitutively active β-Catenin (*β-Catenin*^*fl(ex3)/+*^*; Fgfr3*^*iCreER/+*^) cochlea versus control (*β-Catenin*^*+/+*^*; Fgfr3*^*iCreER/+*^) showing p75 (green), CD44 (red) and Sox2 (blue) reveals multiple clusters of p75+ cells within the IPC region (white arrowheads) (B-C) Quantification of the p75+ and CD44+ cell density in each turn of constitutively active *β-Catenin* cochlea compared to control shows increased p75+ cell density at the expense of CD44+ cell density. (D) Timeline for mating, induction, and cochlear epithelium collection/staining. (E) Immunostaining of whole mount coupled with EdU proliferation assay and lineage tracing for constitutively active *β-Catenin* (*β-Catenin*^*fl(ex3)/+*^*; Fgfr3*^*iCreER/+*^*; Rosa*^*tdTomato/+*^) cochlea versus control shows multiple IPCs (domain outlined between white dashed lines) incorporating EdU proliferation marker (white arrowheads). (F) Quantification of the percentage of recombined IPCs in constitutively active *β-Catenin* cochlea versus control. (G) Quantification of the percentage of EdU+ recombined IPCs in constitutively active *β-Catenin* cochlea versus control shows no EdU+ recombined IPCs in control, while around 12.5% of recombined IPCs incorporated EdU marker in the base and middle turns of constitutively active *β-Catenin* cochlea. Scale bar = 100μm. n = 3, mean ± SE, Student’s t-Test p-value indicated.

To investigate whether β-Catenin is sufficient for inducing ectopic IPC formation in the developing cochlea, we utilized a *β-Catenin*^*fl(ex3)*^ allele that generates constitutively active β-Catenin under the control of Cre recombinase [41]. We crossed this line with *Fgfr3*^*iCreER/+*^ inducible Cre model which is activated in supporting cells upon induction [42] to generate constitutively active β-Catenin mutants (*β-Catenin*^*fl(ex3)/+*^*; Fgfr3*^*iCreER/+*^) and littermate controls (*β-Catenin*^*+/+*^*; Fgfr3*^*iCreER/+*^*)*. Pregnant mice were induced with tamoxifen at E14.5 and cochleae were collected and analyzed at E18.5. Constitutively active β-Catenin cochlea exhibited multiple foci of p75+ cell clusters within the IPC region, yet no ectopic IPC formation was found outside the IPC region (Fig 3A). Some cells around the p75+ foci showed CD44 expression disruption (Fig 3A). Quantification of p75+ cell density showed a significant and modest increase in the basal and middle turn of constitutively active β-Catenin mutants versus controls (27.6 cells versus 20.1 /100 μm and 26.9 cells versus 20.9 cells/100 μm, respectively) (Fig 3B). The density of CD44+ cells decreased in the basal turn of the constitutively active β-Catenin mutant cochlea (11.1 cells/100 μm versus 14 cells/100 μm in control) (n = 3, mean, Student’s t-test p <0.05) (Fig 3C). OPCs in the vicinity of IPC foci were displaced and showed lower CD44 expression but no p75 expression, suggesting that these cells started to lose OPC identity but did not acquire an IPC fate.

To pinpoint the source of p75+ cell clusters in constitutively active β-Catenin mutant cochlea, we crossed the *Rosa*^*tdTomato*^ reporter mouse line to the constitutively active β-Catenin model to generate *β-Catenin*^*fl(ex3)/+*^*; Fgfr3*^*iCreER/+*^*; Rosa*^*tdTomato/+*^ embryos and littermate controls (*β-Catenin*^*+/+*^*; Fgfr3*^*iCreER/+*^*; Rosa*^*tdTomato/+*^). We induced recombination in pregnant females with tamoxifen at E14.5, injected EdU (proliferation marker) at E18.5, then collected embryos 2 hours later (Fig 3D). Recombination efficiency within the IPCs as indicated by tdTomato expression was as follows: 55% in the base, 60.6% in the middle, and 54.3% in the apex (Fig 3E and 3F). In the *β-Catenin*^*fl(ex3)/+*^*; Fgfr3*^*iCreER/+*^*; Rosa*^*tdTomato/+*^ cochlea, EdU marked some of the tdTomato+ IPCs (12.9% (base), 13.1 (middle) and 3.2% (apex)), whereas none were marked in control cochlea (Fig 3E–3G). No EdU incorporation was noticed outside the IPC region and within the organ of Corti, indicating that IPCs are the only supporting cell population showing β-Catenin-induced proliferation. These data indicate that constitutive activity of β-Catenin within supporting cells is sufficient to induce proliferation in IPCs.

### Single cell RNA sequencing of β-Catenin-deficient supporting cells shows loss of IPC identity

To understand the molecular identity of the β-Catenin-deficient supporting cells within the IPC region, we utilized single cell RNA (scRNA) sequencing of cochlear duct cells from dm-cKO (*β-Catenin*^*dm/fl*^*; Fgf20*^*Cre/+*^) P0 pups and littermate controls (*β-Catenin*^*fl/+*^*; Fgf20*^*Cre/+*^). We pooled both cochleae of one control pup at P0 and analyzed 2,830 cells with 58,718 mean reads per cell, and a sequence saturation of 73.5%. Around 87% of the reads were mapped to the mouse genome and a total of 18,757 genes were detected. We analyzed control sample data using unsupervised graph-based clustering in Loupe Browser v.6 (10X genomics) to cluster similar transcriptome signatures from control cochlear cells. We were able to identify unique transcriptomic signatures belonging to specific cell populations based on previously published markers. Hair cells markers such as *Barhl1*, *Pou4f3*, *Pvalb* were enriched in hair cell clusters [43], *Lfng*, *Fgfr3* and *Prox1* [20] were enriched in supporting cells (S3A–S3C Fig). For the top 20 enriched genes per cluster, see Table 1. For the full list of differentially expressed genes, please refer to S1 Table.

**Table 1:** Top 20 enriched genes per population in P0 control cochlear duct.

Mesenchymal cells	Hair cells	Inner phalangeal/Inner border cells	Supporting cells*	Outer sulcus/Claudius cells	Kolliker’s organ cells	Reisner’s membrane cells	Blood/immune cells
*Col3a1*	*Ccl21a*	*Gjb6*	*Fgfr3*	*Fst*	*Crabp1*	*Pi15*	*Ctla2a*
*Pou3f4*	*Pou4f3*	*Gjb2*	*Fam159b*	*Bmp4*	*1500015O10Rik*	*Ttr*	*Ccl4*
*Otor*	*Grxcr1*	*Prss23*	*Prox1*	*A930011G23Rik*	*Adgrg2*	*Otx2*	*C1qc*
*Tbx18*	*Barhl1*	*Ntf3*	*Cep41*	*Gata2*	*Smoc2*	*A930003A15Rik*	*Cdh5*
*Ifitm1*	*Mkrn2os*	*Matn4*	*Lfng*	*Lmo3*	*Pcolce2*	*Slc26a7*	*Cd93*
*Tgfbi*	*Otof*	*Slitrk6*	*Npy*	*Cgnl1*	*Epyc*	*Oc90*	*C1qa*
*Atp1a2*	*Lhfpl5*	*Moxd1*	*Smagp*	*Npnt*	*Cdh4*	*Vmo1*	*Cldn5*
*Bgn*	*Pvalb*	*Hes5*	*Slitrk6*	*Hmga2*	*Tns3*	*Lypd2*	*Ccl3*
*Creb5*	*Tomt*	*Cpxm2*	*Ptprz1*	*Fbln2*	*Chst15*	*Dapl1*	*Kdr*
*Col1a2*	*Rasd2*	*Chst2*	*Hes5*	*Smad6*	*Tecta*	*Rnase1*	*Pf4*
*Apod*	*Ppp1r27*	*Tsen15*	*Emid1*	*Hs3st1*	*Clu*	*Wnt4*	*Fcer1g*
*Pcca*	*Tmem255b*	*Fgf10*	*Tectb*	*Itih5*	*Mt3*	*Prss33*	*Hba-a2*
*Pid1*	*Kcnh7*	*Igfbp3*	*Ppp2r2b*	*Rnd2*	*Rgcc*	*Ntn1*	*Hba-a1*
*Nbl1*	*Thsd7b*	*Fabp7*	*Cyp26b1*	*Adgra3*	*Mia*	*Slc6a15*	*Cxcl2*
*Kctd12*	*Acbd7*	*Tectb*	*Ntf3*	*Ppfibp1*	*Muc15*	*Rnase4*	*Hbb-bs*
*Ccnd2*	*Stmn3*	*Jag1*	*Fzd9*	*Cdo1*	*Bcl2*	*Cndp2*	*Hbb-bt*
*Igfbp2*	*Cxcl14*	*Anxa5*	*Lockd*	*Sfrp1*	*Dkk3*	*Fibin*	*Tyrobp*
*Eva1b*	*Atoh1*	*Sox2*	*Adgrg6*	*Rtn1*	*Twf1*	*Ccdc3*	*Cd34*
*Emcn*	*BC030867*	*4930426D05Rik*	*Sox2*	*Tpbg*	*Efhd1*	*Sgk3*	*C1qb*
*Pam*	*Dlk2*	*Fstl1*	*Nav2*	*Gata3*	*Spock1*	*Fgf9*	*Ccl7*

* Supporting cells include IPCs, OPCs and Deiters’ cells

**Fig 4:**
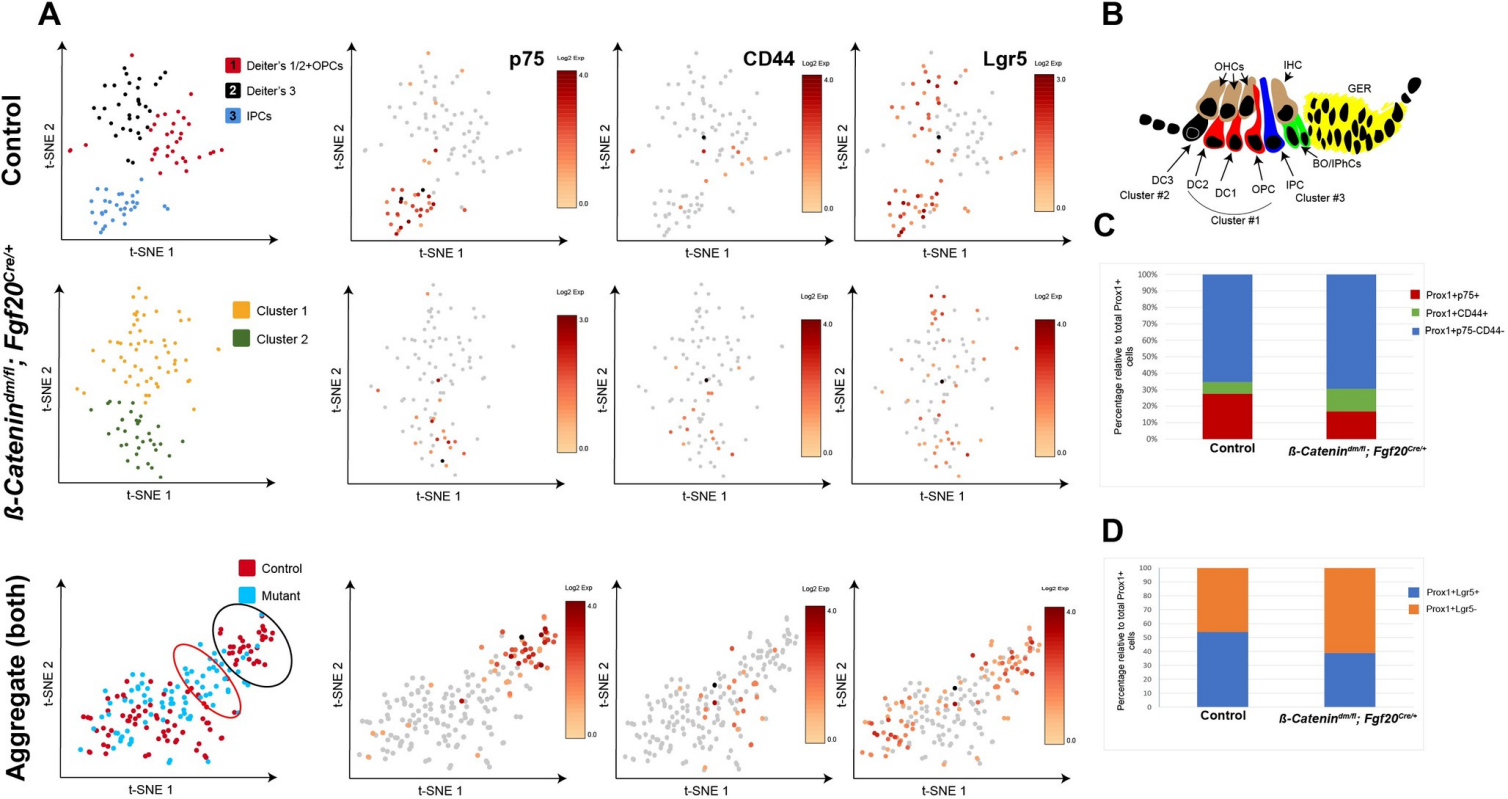
β-Catenin transcriptional activity within supporting cells is required for inner pillar cell gene expression signature. (A) t-SNE plots representing graph-based clustering of scRNA sequencing data from Prox1+ supporting cell population at P0 in control (*β-Catenin*^*fl/+*^*; Fgf20*^*Cre/+*^: top panel), dm-cKO (*β-Catenin*^*dm/fl*^*; Fgf20*^*Cre/+*^; middle panel) and aggregate of both samples (bottom panel) show 3 different cell clusters in control, identified based on known markers of each population (IPCs in blue, 3^rd^ row of Deiters’ cells in black, and OPCs together with 1^st^ and 2^nd^ rows of Deiters’ cells in red). dm-cKO plots (middle panel) shows only 2 cell clusters (color-coded) with decreased p75 expression and increased CD44 expression when compared to control. Upon aggregating both samples (lower panel), control IPCs make a tight cluster expressing p75 (black oval), while cells from dm-cKO are in a transitional zone relative to the rest of Prox1+ cells (red oval) (Loupe Browser v6.0, 10x Genomics). (B) Schematic diagram of organ of Corti cells depicting the 3 different clusters of supporting cells found in control (C) Quantification of the percentage of IPCs (Prox1+p75+), OPCs (Prox1+CD44+), and Deiters’ cells (Prox1+, p75-, and CD44-) relative to total Prox1+ cells in control versus dm-cKO cochlea at P0 showing a decrease in IPCs and gain of OPCs in dm-cKO. (D) Quantification of the percentage of Lgr5+ cells (marker of 3^rd^ row of Deiters’ cells and IPCs) relative to total Prox1+ cells in control versus dm-cKO cochlea at P0.

Using single cell sequencing data from the control sample, we further selected the Prox1+ supporting cell population and re-clustered this population to identify specific subclusters within the supporting cells at P0. Unsupervised graph-based clustering identified 3 different clusters within supporting cells at P0 (Fig 4A, top panel). IPCs formed a separate cluster as indicated by p75 expression, while the other 2 clusters were partially connected. To identify the identity of the other 2 clusters, we examined CD44 (OPC marker) and Lgr5 (marker of 3^rd^ row of Deiters’ cells and IPCs) expression (Fig 4A and 4B). Data showed that the 3^rd^ row of Deiters’ cells (DC3) clustered separately from the 1^st^ and 2^nd^ rows of Deiters’ cells (DC1/2). OPC cells (CD44+ cells) were located within the DC1/2 cluster, indicating that OPCs’ molecular signature is similar to the DC1/2 signature at P0 (Fig 4A). We analyzed differentially expressed genes (DEGs) among the 3 clusters (IPCs, DC3 & DC1/2) and identified enriched genes within each cluster (see Table 2 for the top 20 genes per cluster and S2 Table for the full list) which constitute the molecular signature of each cluster at P0.

**Table 2:** Top 30 enriched genes per supporting cell subcluster in P0 control cochlear duct.

Deiters’ 1/2 + OPCs enriched genes	Deiters’ 3 enriched genes	IPC enriched genes
*Cd74*	*Cdkn1a*	*Npy*
*H2-Aa*	*Fgfr1*	*Wnt5a*
*Egfl6*	*S100a6*	*Ngfr*
*Nr4a3*	*Tbc1d10a*	*Lypd6*
*Tnnt1*	*Nupr1*	*Trps1*
*Lgals3*	*S100a13*	*Npnt*
*Pdzk1ip1*	*Tdg*	*Nrxn3*
*Klf6*	*Sfrp1*	*Hspb2*
*Gstm1*	*Emx2*	*Ccnd1*
*Frzb*	*Tsen15*	*Hs3st6*
*Nfix*	*Rffl*	*Crym*
*Atf3*	*Grtp1*	*Tuba4a*
*Cntn1*	*Fam102b*	*Gpx8*
*Krt8*	*Ddah2*	*2810006K23Rik*
*Dbi*	*Setbp1*	*Fsd1l*
*Bcl2*	*Clu*	*Cryab*
*Car14*	*Fam204a*	*Lama2*
*Krt18*	*Agl*	*Mdm1*
*Fam46a*	*Rbm8a*	*Gm13782*
*Lsamp*	*Prss23*	*Etv4*
*Cdkn1c*	*Klf4*	*Pxmp2*
*Dusp1*	*S100a1*	*Cadm1*
*Glul*	*Lhfp*	*Snx22*
*Junb*	*Hist3h2a*	*Lrba*
*Nr4a1*	*Gas1*	*Snrnp25*
*Igsf3*	*Bend4*	*Map4*
*Gm2a*	*Syt6*	*Cdk2ap1*
*Ptn*	*Ypel2*	*Ucp2*
*Egr2*	*Ivns1abp*	*Tmem159*
*Rassf10*	*Ncor1*	*Sdf2l1*

We next analyzed P0 dm-cKO cochlear cells using the same methods mentioned above. We pooled both cochleae from one pup and were able to analyze a total of 2,991 cells, with mean reads of 50,736 reads per cell and sequence saturation of 72.1%. Prox1+ supporting cell re-clustering showed only 2 clusters compared to 3 clusters in control (Fig 4A, middle panel). Mapping p75, CD44 and Lgr5 expression within these 2 clusters showed that Cluster #2 included some cells expressing the IPC marker (p75), others expressing OPC marker (CD44) and a third group of cells expressing both markers (Fig 4A, middle panel). To identify potential shifts in molecular signatures in dm-cKO versus control single cell transcriptomes, we integrated (aggregated) both control and dm-cKO single cell sequencing data from Prox1+ cells (Fig 4A, lower panel). p75+ cells from control sample formed a tight cluster (black oval) while p75+ cells from dm-cKO showed lower p75 expression and shifted towards the CD44+ cell cluster (red oval), indicating a transcriptome signature shift deviating away from the control IPC signature.

Next, we quantified the percentage of p75+ cells (IPCs), CD44+ cells (OPCs) and p75-negative, CD44-negative cells (Deiters’ cells) to the total Prox1+ cells in dm-cKO and control cochleae (Fig 4C). IPCs represented 27.5% of the total Prox1+ cells in control cochlea versus 16.8% in dm-cKO cochleae, whereas OPCs represented 7.1% of the total Prox1+ cells in control versus 13.7% in dm-cKO cochleae. p75-negative, CD44-negative cells (Deiters’ cells) represented 65.3% in control versus 69.5% in dm-cKO (Fig 4C). These data suggest a loss of IPC signatures and gain of OPC signatures in dm-cKO cochlea. The percentage of Prox1+ cells expressing *Lgr5* (marker for both IPCs and DC3 cells) was lower in dm-cKO (38.9%) compared to control (54%) cochleae, confirming the loss of IPC signatures in dm-cKO cochleae (Fig 4D).

**Fig 5:**
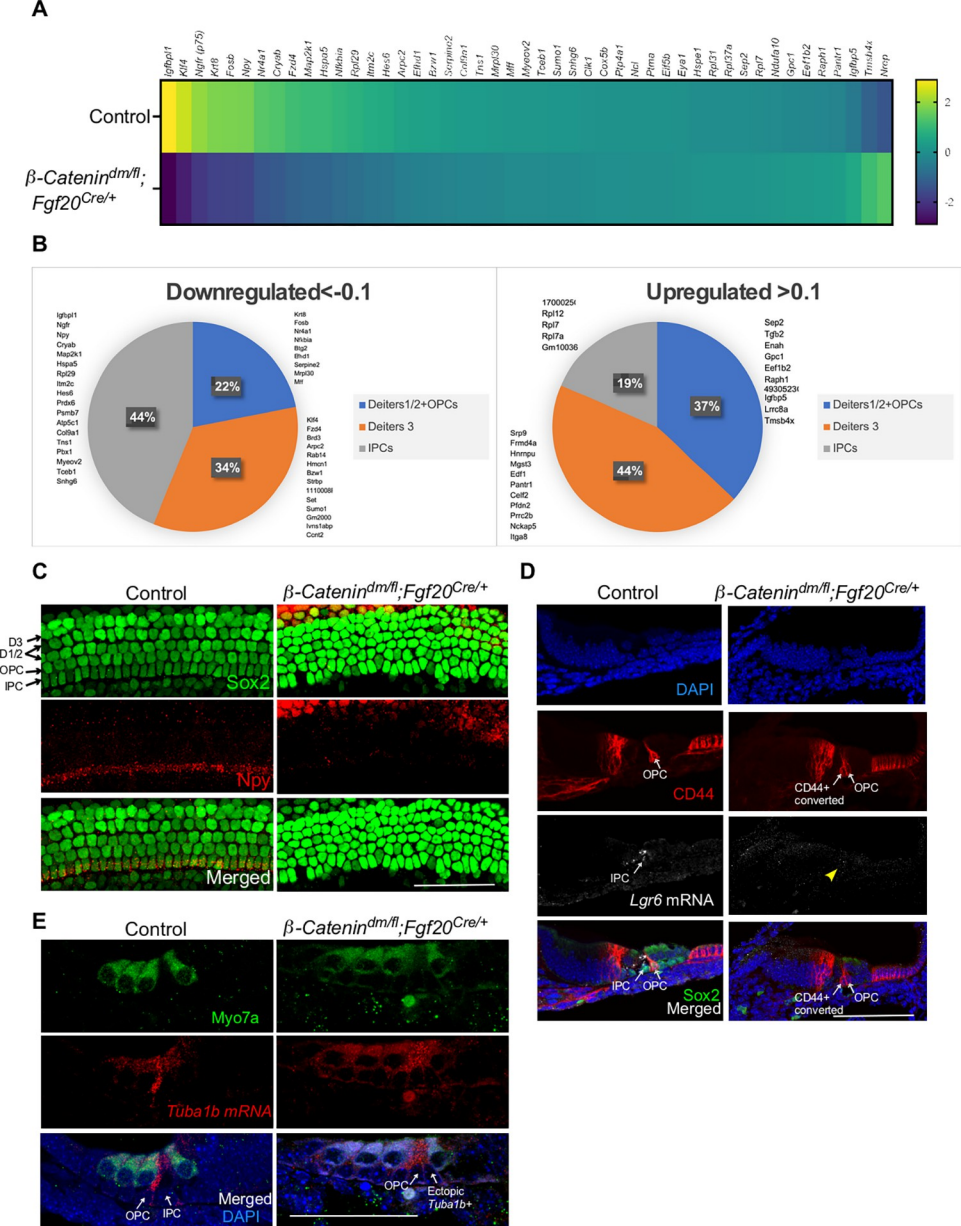
β-Catenin transcriptional activity regulates gene expression within supporting cells to establish inner pillar cell gene expression signature. (A) Heat map of differentially expressed genes within Prox1+ cells at P0 in control (*β-Catenin*^*fl/+*^*; Fgf20*^*Cre/+*^) versus dm-cKO (*β-Catenin*^*dm/fl*^*; Fgf20*^*Cre/+*^) showing top up-and downregulated genes (Loupe Browser v6.0, 10x Genomics). (B) Pie charts of the percentage of differentially expressed genes that are cross-referenced to the molecular signatures of the 3 identified clusters of supporting cells at P0: IPCs, 3^rd^ row of Deiters’ cells, and OPCs along with 1^st^ and 2^nd^ rows of Deiters’ cells. The biggest percentage of downregulated genes belongs to the IPC gene signature. (C) Validation of IPC identity loss in dm-cKO using whole mount immunostaining of E18.5 cochlear epithelium (basal turn) showing Npy expression (IPC marker) in red along with Sox2 (supporting cells marker) in green. Cochleae from dm-cKO mice shows lack of Npy staining when compared to control. (D) Fluorescence in-situ hybridization for IPC marker *Lgr6* mRNA along with immunostaining for CD44 at P0 showing loss of Lgr6 expression in dm-cKO (yellow arrowhead) (F) Fluorescence in-situ hybridization for potential OPC marker (*Tuba1b*) along with immunostaining for Myo7a (HC marker) in P0 showing gain of *Tuba1* expression in IPC region in dm-cKO. Scale bar = 100μm.

We next analyzed differentially expressed genes (DEGs) in Prox1+ cells in control and dm-cKO cochleae (Fig 5A) and cross referenced the DEGs to the gene signatures of each of the 3 clusters of supporting cells from the control sample (IPCs, DC1/2, DC3) (Fig 5B). Forty-four percent of downregulated genes in dm-cKO belong to the IPC signature, including *Igfbpl1*, *p75*, *Npy* and *Cryab*, while 44% of upregulated genes in dm-cKO belong to the DC3 signature, including *Srp9*, *Frmd4a*, *Hnrnpu* and *Mgst3*. These results suggest a loss of the IPC signature upon deletion of β-Catenin transcriptional activity. For the top DEGs, please refer to Table 3.

**Table 3:** Top differentially expressed genes within Prox1+ cells in dm-cKO versus control at P0.

Gene symbol	Control Average	dm-CKO Average	Log2 Fold Change	P-Value
*Igfbpl1*	1.007729	0.126634	-2.89193	0.001825
*Klf4*	1.64469	0.29548	-2.43587	0.000696
*Ngfr*	1.435538	0.358797	-1.96955	0.087231
*Krt8*	3.945354	1.150261	-1.76998	0.01245
*Fosb*	2.110526	0.622618	-1.74491	0.00412
*Npy*	13.88004	4.231692	-1.71259	0.389169
*Nr4a1*	1.758772	0.675382	-1.3677	0.124379
*Cryab*	20.13556	8.231222	-1.29089	0.100534
*Fzd4*	1.197866	0.548748	-1.11167	0.396318
*Map2k1*	1.140825	0.559301	-1.01488	0.761674
*Hspa5*	1.625676	0.802017	-1.01038	0.362721
*Nfkbia*	1.597155	0.854781	-0.89423	0.881458
*Brd3*	1.036249	0.559301	-0.87738	1
*Rpl29*	34.19623	19.8077	-0.7889	0.363119
*Itm2c*	1.06477	0.675382	-0.64872	1
*Hes6*	1.036249	0.675382	-0.6099	1
*Prdx6*	1.416525	0.970862	-0.54056	1
*Btg2*	6.901992	4.790993	-0.52699	1
*Arpc2*	1.273921	0.928651	-0.45199	1
*Rab14*	1.06477	0.791464	-0.42316	1
*Hmcn1*	1.045756	0.780911	-0.4165	1
*Psmb7*	1.017236	0.759805	-0.41597	1
*Efhd1*	1.94891	1.487952	-0.38766	1
*Bzw1*	1.06477	0.823122	-0.3673	1
*Atp5c1*	3.089735	2.458814	-0.32927	1
*Serpine2*	2.652418	2.163334	-0.29369	1
*Col9a1*	1.863348	1.561822	-0.25379	1
*Tns1*	1.910882	1.604033	-0.25173	1
*Mrpl30*	2.034471	1.74122	-0.22406	1
*Gpc1*	1.97743	2.289968	0.20992	1
*Eef1b2*	10.88537	12.65287	0.215523	1
*Raph1*	1.045756	1.245236	0.249497	1
*Hnrnpu*	1.026743	1.22413	0.251276	1
*Mgst3*	4.097464	4.959839	0.273791	1
*Edf1*	1.464059	1.772879	0.27385	1
*4930523C07Rik*	2.091513	2.553789	0.286005	1
*Pantr1*	2.58587	3.18696	0.299512	1
*Celf2*	2.671432	3.471887	0.375866	1
*Pfdn2*	0.979208	1.287448	0.39117	1
*Prrc2b*	0.922167	1.245236	0.429203	1
*Nckap5*	1.273921	1.762326	0.464597	1
*Itga8*	0.770057	1.086943	0.491983	1
*Igfbp5*	16.00007	23.55396	0.556186	1
*Lrrc8a*	0.655974	1.055285	0.678024	1
*Tmsb4x*	4.848507	10.31013	1.085606	0.389169
*Nrep*	0.427809	1.118602	1.367	0.100534

We next analyzed enriched gene ontologies within upregulated and downregulated gene sets in dm-cKO vs control Prox1+ supporting cells using the Database of Annotation, Visualization, and Integrated Discovery (DAVID) [44,45]. Within the downregulated genes, we found genes associated with canonical Wnt signaling (*fzd4* and *klf4*), MAPK signaling pathway genes (*p75*, *Nr4a1*, and *Map2k1*), and few transcription factors (*Hes6*, *Nr4a1*, *Fosb*, and *Klf4*). Enriched gene ontologies within upregulated genes included multiple genes involved in cell differentiation (*Enah*, *Itga8*, *Hnrnpu*, *Lrrc8a*, *Edf1* and *Prrc2b)* (S4A and S4B Fig). To validate the loss of IPC signature in dm-cKO supporting cells, we immunostained cochlear samples from both models against a recently identified IPC marker *Npy* [43], and found it to be significantly reduced in all turns of dm-cKO cochlea (Fig 5C). Through fluorescence *in situ* hybridization, we validated that another IPC maker (*Lgr6*) was downregulated in dm-cKO supporting cells (Fig 5D). Lastly, we found ectopic expression of the OPC marker *Tuba1b* in the IPC region in dm-cKO cochleae (Fig 5E), providing additional evidence for the IPC-to-OPC identify shift.

Since we observed multiple differentially expressed genes that belong to the Deiters’ cells, we excluded p75+ and CD44+ population from the supporting cell transcriptomes, then performed differential gene expression analysis on Prox1+, p75-negative, and CD44-negative cells in control and dm-cKO cochleae. The top downregulated genes in dm-cKO DCs were *Atf3*, *Klf4*, *Fosb*, *Krt8*, *Nr4a1*, *Egr1*, *Serpine2*, *Bcl2*, *Efhd1*, and *Dtymk*. Conversely, the top upregulated genes were *Gm10036*, *Gadd45gip1*, *Snhg20*, *Nckap5*, *Ndufa10*, *Cox5b*, *Tceb1*, *Sep2*, *Asnsd1*, and *Rpl7*. The complete list of DEGs is listed in S3 Table. These data suggest that ablation of the β-Catenin transcriptional pathway may also alter Dieters’ cell gene expression.

### β-Catenin cell autonomous role in establishing IPC identity

**Fig 6:**
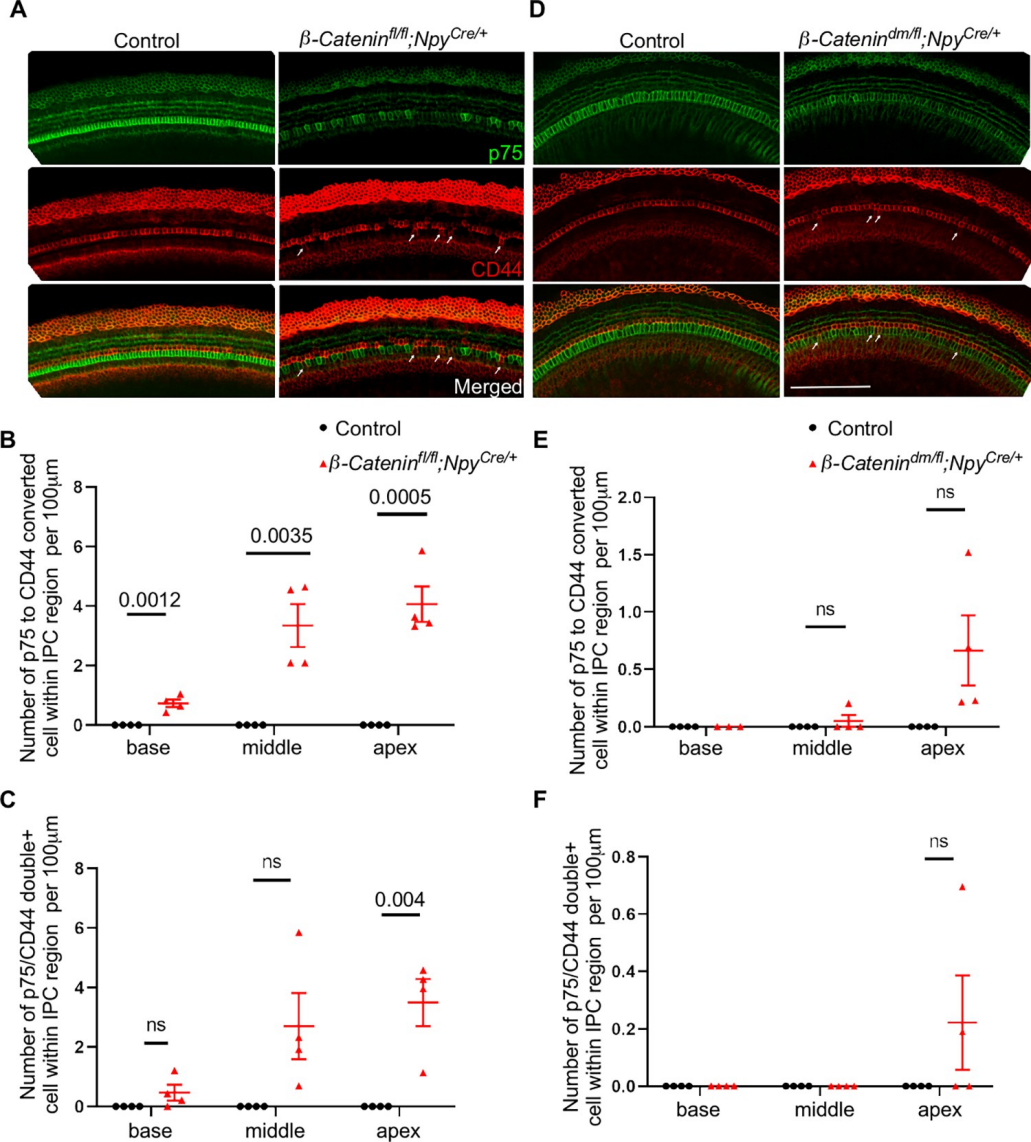
β-Catenin works in a cell-autonomous manner to maintain IPC identity as shown in IPC-specific *β-Catenin* deletions. (A) Immunostaining of whole mount E18.5 cochlear epithelium (basal turn) for IPC marker p75 (green) and OPC marker CD44 (red) from IPC-specific complete *β-Catenin* deletion (*β-Catenin*^*fl/fl*^*; Npy*^*Cre/+*^) versus control showing a subset of cells within IPC region lacking p75 staining and acquiring CD44+ staining in IPC-specific *β-Catenin* cKO (white arrows). (B-C) Quantification of the number of p75 to CD44 converted cells and p75/CD44 double positive cells within the IPC region per 100μm in IPC-specific complete *β-Catenin* deletion versus control. (D) Immunostaining of E18.5 cochlear epithelium (basal turn) for the IPC marker p75 (green) and the OPC marker CD44 (red) in IPC-specific *β-Catenin* transcriptional deletion (*β-Catenin*^*dm/fl*^*; Npy*^*Cre/+*^) versus control showing consistent results as in A. (E-F) Quantification of the number of p75-to-CD44 converted cells and p75/CD44 double positive cells within the IPC region per 100 μm in IPC-specific *β-Catenin* transcriptional deletion versus control. Scale bar = 100μm. n = 4, mean±SE, Student’s t-Test p-value indicated.

To investigate if β-Catenin functions in a cell-autonomous or non-cell-autonomous manner to establish IPC identity, we utilized an *Npy*^*Cre*^ mouse line to delete β-Catenin from IPCs, as *Npy* expression is restricted to IPCs and is expressed as early as E16.5 [43]. Upon crossing the *Npy*^*Cre*^ line with a *Rosa*^*tdTomato*^ line, tdTomato expression (as a marker of Cre recombination) was restricted to IPCs and glial cells in the cochlea at P0 (S5A Fig). To confirm IPC-specific deletion of β-Catenin, we generated an IPC-specific β-Catenin conditional knockout along with the tdTomato reporter gene (*β-Catenin*^*fl/fl*^*; Rosa*^*tdTomato/+*^*; Npy*^*Cre/+*^). Analysis of P1 cochlea from this conditional knockout showed reduced levels of β-Catenin in the majority of IPCs (S5B Fig). To test the effects of IPC-specific β-Catenin deletion, we generated and analyzed conditional mutants using both *β-Catenin*^*fl*^ and *β-Catenin*^*dm*^ alleles. Immunostaining of whole mount preparation of P1 cochlear epithelium from *β-Catenin*^*fl/fl*^*; Npy*^*Cre/+*^ mouse showed that cells within the IPC region lost p75 expression and gained CD44 expression (Fig 6A–6C). While the overall p75 levels were markedly reduced, only a few IPCs were double positive for CD44 and p75 (S5C Fig). Interestingly, we noted more converted and double positive cells in the apex compared to the base, suggesting some spatial differences on pillar cell fate change. Although the IPC-specific β-Catenin transcriptional deletion cochlea (*β-Catenin*^*dm/fl*^*; Npy*^*Cre/+*^) did not show statistically significant changes, a trend of reduction in p75 expression and occasional ectopic CD44+ cells were observed (Fig 6D–6F). Overall, our results strongly suggest a cell-autonomous role of β-Catenin in establishing IPC identity.

## Discussion

Supporting cells serve multiple crucial roles in the mammalian cochlea, contributing to the structural integrity of organ of Corti during sound stimulation and maintaining the optimal micro-environment required for normal hair cell function [3,46,47]. Pillar cells are supporting cells required to form the tunnel of Corti, which is unique to the mammalian cochlea and is required for normal hearing [16,22,48]. FGF8-FGFR3 signaling pathway has been implicated for pillar cell development [22–27], but the mechanism of IPC versus OPC fate determination is still unknown. This study shows multiple lines of evidence that β-Catenin transcriptional activity is required for IPC identity.

**Fig 7:**
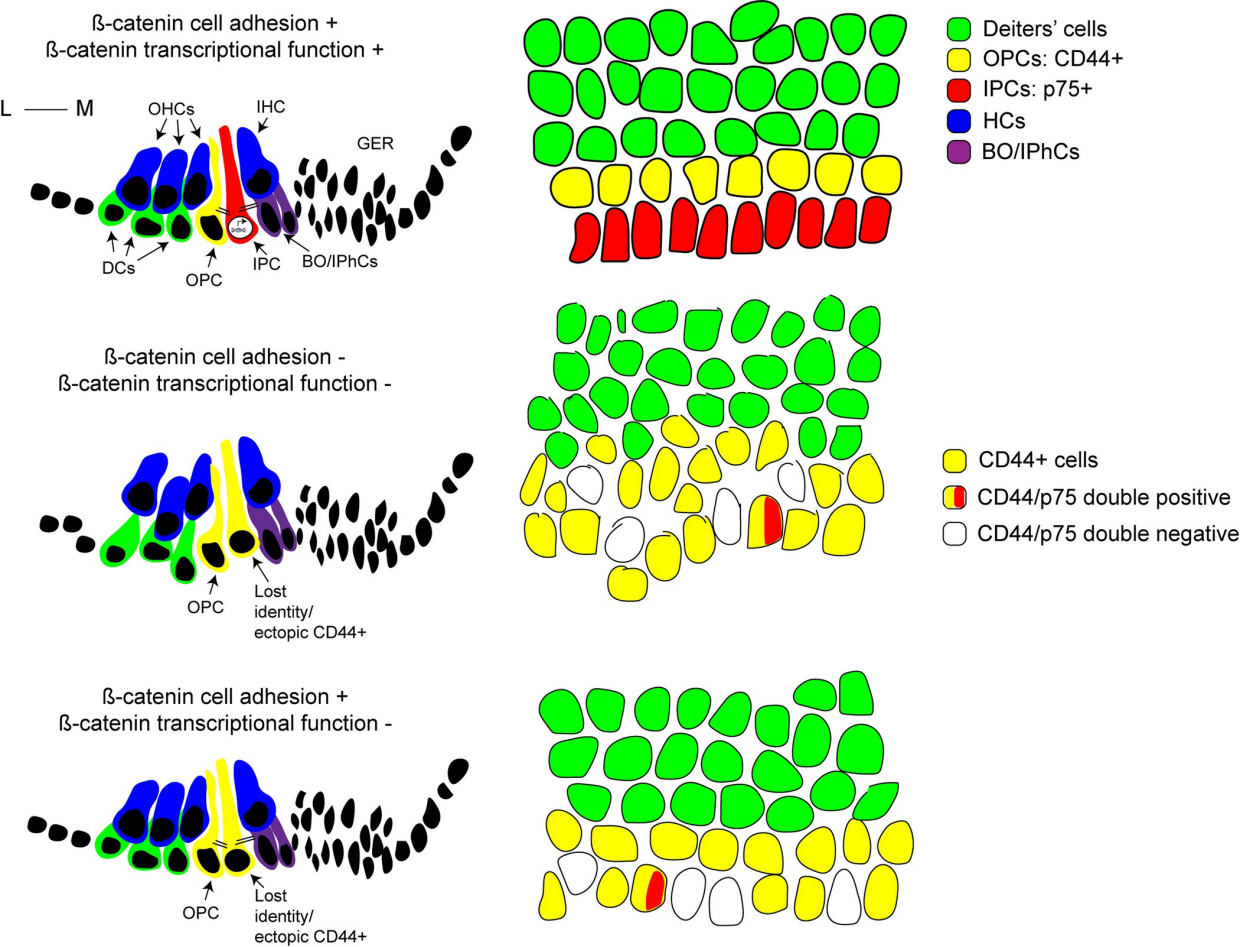
A schematic diagram for β-Catenin transcriptional versus cell adhesion function in supporting cell development. Intact β-Catenin cell adhesion and transcriptional functionalities during supporting cell development are required for normal cell arrangement and establishment of IPC identity (top panel). Lack of both β-Catenin functionalities causes disruption in supporting cell arrangement, disruption of medial-lateral compartment as well as loss of IPC identity (middle panel). Lack of β-Catenin transcriptional function only, while cell adhesion function is intact, causes loss of IPC identity, with subsets of IPCs either expressing OPC markers, expressing both IPC and OPC markers, or neither, indicating lost IPC identity.

Using two independent β-Catenin deletion models (complete deletion and transcriptional deletion) along with multiple Cre drivers, we show that lack of β-Catenin causes cells within the IPC region to lose IPC signature genes (such as p75, Npy, and Lgr6) and gain OPC genes (such as CD44 and Tuba1b) (Fig 7) indicating that the β-Catenin transcriptional role is involved in establishing IPC versus OPC identity. It is not clear why cells in the IPC region lose p75 expression while gaining expression of CD44. We postulate that the OPC fate might be the default fate for pillar cells, and only when β-Catenin transcriptional activity is turned on, the IPC fate is established. The phenotype of ectopic OPCs was more evident in the complete deletion model compared to the transcriptional deletion model (Fig 1I and 1J), suggesting that cell adhesion may also play a role in pillar cell development. We have observed an increase in the density of Prox1+ supporting cells in these models that is not accompanied by change in hair cell density, indicating that changes in cochlear duct length is not contributing to the phenotype. Immunostaining for the proliferation marker Ki67 did not detect a difference in mutant cochleae, thereby ruling out a change in proliferation of supporting cell progenitors. We postulate that there might be a fate change from lesser epithelial ridge (LER) cells to supporting cells following β-Catenin deletion, which warrant further examination in future experiments. Lineage tracing of β-Catenin-deficient cells showed a loss of p75 and gain of CD44 expression from a subset of recombined cells within the IPC region in both complete and transcriptional deletion models. Not all recombined cells showed this phenotype, suggesting that β-Catenin activity may not be the sole determinant of IPC fate. Nevertheless, our findings confirm the requirement of β-Catenin transcriptional role for IPC identity.

Using a constitutively active β-Catenin model, we tested whether β-Catenin is sufficient for inducing IPC fate. Data show no ectopic IPCs outside their respective cochlear domain, but that some IPCs proliferated to create clusters of IPCs (Fig 3). This data shows that IPCs are capable of β-Catenin-induced proliferation during development, yet β-Catenin is not sufficient to induce IPC fate in supporting cells. Our data aligns with recently published work showing that β-Catenin stabilization increases proliferation in cochlear epithelial cells [49].

In addition to using p75 and CD44 as markers for IPCs and OPCs, respectively [50,51], we analyzed the molecular signature of pillar cells in β*-*Catenin transcriptional deletion model to confirm the loss of IPC identity and to identify targets downstream β-Catenin during supporting cell development. Single cell transcriptomic analysis at P0 showed that supporting cells lacking the β-Catenin transcriptional function failed to establish the unique IPC molecular signature as identified in the controls. Although we utilized one biological sample per condition for the scRNA sequencing experiment, we have validated a subset of DEGs using immunostaining and *in situ* hybridization to support IPC identity loss. Analyzing supporting cells from the *β-Catenin* transcriptional deletion model, we observed a significant loss of IPC specific genes and gain of other supporting cells-specific genes including Deiters’ and/or OPC marker genes. Downregulated genes in *β-Catenin* deficient supporting cells did not show canonical Wnt target genes such as *Axin2*, *Lgr5* or *Sp5*, which may be due to the late time point of the scRNA sequencing sample (P0) relative to the time of IPC specification and differentiation (around E15) [23–25]. A valid follow up experiment is to investigate the expression of Wnt target genes within the supporting cells from *β-Catenin* deletion models at earlier time points (E15.5–16.5). Nevertheless, P0 single cell transcriptome analysis shows the ultimate impact of *β-Catenin* deletion on pillar cell fates.

Finally, we limited *β-Catenin* deletion to IPCs to determine its cell autonomous role in IPC fate establishment. IPC-specific conditional *β-Catenin* deletion resulted in a similar phenotype of IPC to OPC marker conversion within IPCs. Notably, this phenotype was more evident in the apex than in the middle and basal turns. We suggest that pillar cell specification follow a basal to apical gradient similar to hair cell differentiation. Basal cells may have already established IPC identity at the time of Cre-mediated *β-Catenin* deletion. This may explain why we found fewer p75 to CD44 conversions in the basal and middle turns compared to the apex. Nevertheless, the occurrence of converted cells and CD44/p75 double positive cells in all turns in the cochlea suggests a cell autonomous role of β-Catenin in IPC identity establishment.

While this work has shown β-Catenin transcriptional activity requirement for IPC fate, there are still multiple questions that need to be addressed. First, which Wnt(s) is required for IPC fate? It has been shown that *Wnt5a* and *Wnt7a* are expressed in cochlear duct at E14.5 [52,53] and play a role in regulating planar cell polarity. However, it is not clear if these ligands also contribute to canonical Wnt signaling activity in supporting cells. Deleting Wnt ligands during pillar cell specification could help answer this question. Second, how is Wnt receptor expression spatially and temporally regulated to ensure restricted Wnt signaling activity to IPCs? A few Wnt receptors, such as *Fzd1* and *Fzd2*, are expressed within the supporting cells during development (reviewed in [54]) and may contribute to IPC fate establishment. Third, does canonical Wnt/β-Catenin signaling interact with FGF8/FGFR3 signaling to establish pillar cell fate? All these questions are unanswered and further research is needed to address them.

One of the most significant applications of Wnt/β-Catenin signaling role in IPC fate is for hair cell regeneration. Activation of Wnt signaling within IPC was sufficient to drive proliferation, as shown in this study. Since proliferation is a prerequisite for regeneration after damage in avian cochlea [55], activating proliferation in established IPCs can be the source of regenerating cells that can be further programmed to hair cells through gene manipulation [56–58]. It is still unclear what is the molecular signature of proliferating IPCs upon Wnt activation, and whether these cells carry the programming potential to become hair cells.

## Conclusion

This work provides a thorough comparison of supporting cell phenotypes in *β-Catenin* complete deletion models versus *β-Catenin* transcriptional deletion models (Fig 7). We show using multiple lines of evidence that β-Catenin transcriptional activity during supporting cell development is required to establish IPC identity. Postnatal roles of β-Catenin in IPC maturation and maintenance remain to be further tested with similar experiments involving cochlea from later postnatal stages.

## Methods

### Ethics statement

This study was carried out in accordance with the recommendations in the Guide for the Care and Use of Laboratory Animals of the National Institutes of Health. All protocols were approved by the Animal Care and Use Committees at Stanford University, and University of Nebraska Medical Center (IACUC numbers 18606 and 16-005-02-EP, respectively).

### Animals

*Emx2*^*Cre*^ [38], *β-Catenin*^*fl*^ [35], *Fgf20*^*Cre*^ [37], *β-Catenin*^*dm*^ [36], *Lgr5*^*CreERT2*^ [59], *Rosa*^*tdTomato*^ [40], *β-Catenin*^*fl(ex3)*^ [41], *Fgfr3*^*iCreER*^ [42], and *Npy*^*Cre*^ [60] were previously described. For experiments, mice were time-mated, and females were checked daily for presence of post-copulatory vaginal plugs, and if present, the developmental stage of the litter was considered E0.5. At the time points of interest, pregnant female mice were euthanized, and embryos were collected. Mice were maintained on a 129X1/SvJ;C57BL/6J mixed background.

### Immunostaining

For whole mount immunostaining, embryos from different time points and groups were collected, inner ears were dissected in cold 1X Phosphate-buffered saline (PBS), fixed with 4% paraformaldehyde (PFA) overnight at 4°C then washed with 1X PBS three times. Samples were blocked in 1X PBS containing 0.5% Triton and 5% normal donkey serum. Samples were then incubated in primary antibody overnight at 4°C, then washed with 1X PBS three times. Samples were incubated with a secondary antibody for 2 hours at room temperature then washed, mounted in 95% glycerol or DAPI mounting medium, cover-slipped, and imaged using a Zeiss LSM 700 confocal microscope. For cryosection immunostaining, PFA-fixed samples were washed in 10%, 20%, and 30% sucrose solutions for 1 hour each at 4°C, then embedded in optimum cutting temperature medium (OCT), frozen on dry ice, and stored at −80°C until further processing. OCT-embedded samples were sectioned horizontally at 10μm thickness using a cryostat, then sections were mounted and left to dry at room temperature overnight. Primary antibodies/stains used: Phalloidin (R&D Systems, 1:40), Prox1 (Covance, 1:250), Myo6 (Proteus, 1:200), Myo7a (Proteus, 1:200), CD44 (DSHB, 1:50), p75 (MilliporeSigma, 1:1000), E-cadherin (Cell Signaling Technology, 1:200), Sox2 (SantaCruz, 1:200), Npy (ImmunoStar, 1:100), β-catenin (Cell Signaling, 1:200), and Ki67 (Abcam, 1:500).

### Proliferation assay

Pregnant females were injected with Ethynyl deoxyuridine (EdU) at 400μg/g body weight two hours ahead of collecting the embryos. Embryos were collected and prepared for cryosectioning. Staining for incorporated EdU along was performed according to Click-iT EdU Cell Proliferation Kit (Invitrogen C10340) manufacturer protocol. Stained sections were then imaged on a Zeiss LSM 700 confocal microscope.

### Single cell RNA sequencing

A P0 pup was euthanized, and cochleae were dissected in sterile cold HBSS, then placed in Dispase (1U/mL) in DMEM/F-12 (Stem cell Technologies) for 15 min at 37°C. Cochlear ducts from both sides were dissected and pooled into a single tube, incubated in 0.25% trypsin-EDTA for 15 min at 37°C with gentle trituration every 5 min, followed by trypsin inactivation by adding an equal volume of DMEM/F-12. Dissociated cells were then passed through a 30μm strainer, pelleted at 300g and then resuspended in 100μl of cold PBS supplemented with 1% fetal bovine serum (FBS, Gibco: 26140079). Single cells were captured and lysed, and mRNAs were reverse transcribed into cDNAs using a 10X Genomics Chromium Controller at University of Nebraska Medical Center Sequencing core. cDNA libraries were prepared using Chromium Single Cell 3′ Reagents according to the manufacturer’s instructions. Libraries were sequenced on an Illumina NextSeq. Sequences were aligned to the Ensembl mouse MM10 assembly using Cell Ranger 2.1.1 analysis software (10× Genomics). Processing of the Cell Ranger output data was done with Loupe Browser v.6 (10X Genomics). Gene expression-based clustering information, including t-SNE projections and differential gene expression were done utilizing Loupe Browser v.6. The raw data from the scRNA sequencing is deposited in gEAR database and can be accessed through the following link: https://umgear.org/index.html?multigene_plots=0&layout_id=d4505add&gene_symbol_exact_match=1&gene_symbol=sox2

### RNA fluorescence in situ hybridization (FISH)

Cryosections (10μm) were used for RNA FISH analysis following the manufacturer’s protocol (Molecular instruments, HCR RNA-FISH protocol for fresh frozen [61].

### Quantification and statistics

Data analysis was done using Image J software Version 1.53n [62]. To measure HC and SC density, at least 400μm regions of the base, middle, and apex of whole mount immunostained cochleae were counted and normalized to 100μm. Two independent investigators performed manual counts blindly after separation of individual channels, selection of images/regions without artifacts, conversion to 8-bit images, manually setting of the threshold to reduce background, selection of the region length/area to measure, then manually counting the positive cells using ImageJ cell counter tool. Cells were called positive for a specific staining based on signal to noise ratio. Cell counts were averaged across manual counts and presented on graphs. For each experiment, the numbers of samples (n) are indicated. The *P* value for difference between samples was calculated using multiple unpaired two-tailed Student’s t-test, and *P* < 0.05 was considered as significant. False Discovery Rate (FDR) was used to adjust for multiple comparisons using two-stage step-up. For ScRNA sequencing data analysis, Cell Ranger 2.1.1 analysis software (10X Genomics) was used.

## Supporting information

S1 Fig β-Catenin deletion does not alter proliferation of supporting cell progenitors. (A) Immunostaining of whole mount E14.5 cochlear epithelium for the proliferation marker Ki67 (green) and the prosensory marker Sox2 (red) from *β-Catenin* deletion (*β-Catenin*^*fl/fl*^*; Emx2*^*Cre/+*^) versus control showing scarce proliferating cells within the prosensory domain (PD) in both conditions. Most proliferating cells are located within the greater epithelial ridge (GER) (B) Quantification of Ki67/Sox2 double-positive cells within the prosensory domain per 100μm. Bar on graph is mean±SE. Scale bar = 50μm.
(TIF)

S2 Fig Validating β-Catenin deletion from supporting cells in *Lgr5*^*CreERT2/+*^ model. (A) Immunostaining of the basal turn whole mount E16.5 cochlear epithelium from *β-Catenin*^*fl/fl*^*; Lgr5*^*CreERT2/+*^*; Rosa*^*tdTomato/+*^ cochlea (full deletion) compared to control 48 hours post induction with tamoxifen (E14.5), showing staining for β-Catenin (white) along with tdTomato fluorescence (red) in cells with *Lgr5*^*CreERT2*^ recombination. The majority of IPCs along with other supporting cells shows loss of β-Catenin staining (yellow arrow heads), but a few supporting cells still show some β-Catenin expression (green arrow heads). (B) Immunostaining from *β-Catenin*^*fl/dm*^*; Lgr5*^*CreERT2/+*^*; Rosa*^*tdTomato/+*^ cochlea (transcriptional deletion) showing β-Catenin expression that is similar to controls. Scale bar = 100μm.
(TIF)

S3 Fig Identifying different cell populations and enriched genes per population in P0 control cochlear cells using single cell RNA sequencing and clustering. (A) t-SNE plot representing graph-based clustering of cochlear cells from P0 control (*β-Catenin*^*fl/+*^*; Fgf20*^*Cre/+*^) showing 8 different cell clusters (color-coded) identified based on known markers per population. (B) t-SNE plots representing Prox1+ expression levels within the supporting cell population in P0 control cochlear cells. (C) Differentially expressed genes (DEGs) within each identified cluster from (A) showing level of enrichment of each gene within each cluster (Loupe Browser v6.0, 10x Genomics).
(TIF)

S4 Fig (A-B) Graphs representing enriched gene ontologies within differentially expressed genes in dm-cKO versus control Prox1+ cells (P values are shown on the x-axis).
(TIF)

S5 Fig Validating IPC-specific β-Catenin deletion mouse model. (A) Immunostaining of whole mount P0 cochlear epithelium from *β-Catenin*^*fl/+*^*; Npy*^*Cre/+*^*; Rosa*^*tdTomato/+*^ cochlea showing p75 (green), tdTomato (red) and CD44 (blue) showing restricted Cre activity within IPCs. (B) Immunostaining of whole mount P1 cochlear epithelium from IPC-specific complete β-Catenin deletion (*β-Catenin*^*fl/fl*^*; Npy*^*Cre/+*^*; Rosa*^*tdTomato/+*^) cochlea showing β-Catenin (green), tdTomato (red) showing efficient β-Catenin deletion within the IPCs. (C) Immunostaining of whole mount P1 cochlear epithelium from IPC-specific transcriptional β-Catenin deletion (*β-Catenin*^*fl/dm*^*; Npy*^*Cre/+*^*; Rosa*^*tdTomato/+*^) cochlea showing p75 (green) and CD44 (red) showing examples for p75/Cd44 double-positive cells in IPC region (blue arrowhead) and Cd44+ ectopic cells (white arrowhead). Scale bar = 100μm unless otherwise specified.
(TIF)

S1 Table Differentially expressed genes in different cell populations identified in control P0 cochlear duct cells. (CSV)

S2 Table Differentially enriched genes among the 3 clusters (IPCs, DC3 & DC1/2) in control P0 cochlear duct. (CSV)

S3 Table Differentially expressed genes in dm-cKO versus control cochlear Deiters’ cells. (CSV)
