# Supplementary figures and images for: β-Catenin transcriptional activity is required for establishment of inner pillar cell identity during cochlear development
Source: PLoS Genet. 2023 Aug 28;19(8):e1010925. doi: 10.1371/journal.pgen.1010925 (PMC10491406; doi:10.1371/journal.pgen.1010925)

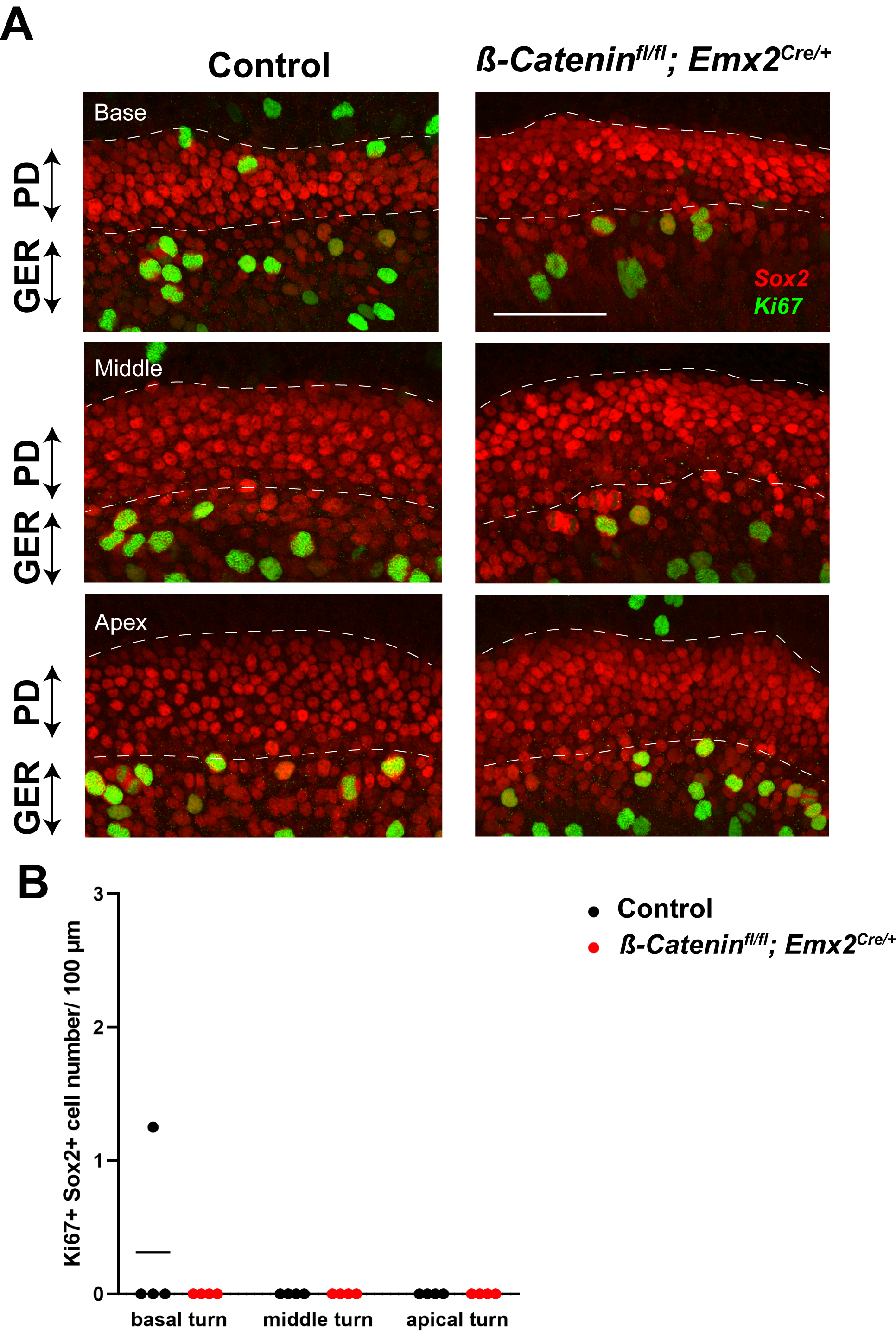

Supplement: S1 Fig — (A) Immunostaining of whole mount E14.5 cochlear epithelium for the proliferation marker Ki67 (green) and the prosensory marker Sox2 (red) from β-Catenin deletion (β-Cateninfl/fl; Emx2Cre/+) versus control showing scarce proliferating cells within the prosensory domain (PD) in both conditions. Most proliferating cells are located within the greater epithelial ridge (GER) (B) Quantification of Ki67/Sox2 double-positive cells within the prosensory domain per 100μm. Bar on graph is mean±SE. Scale bar = 50μm. (TIF) [file pgen.1010925.s001.tif]

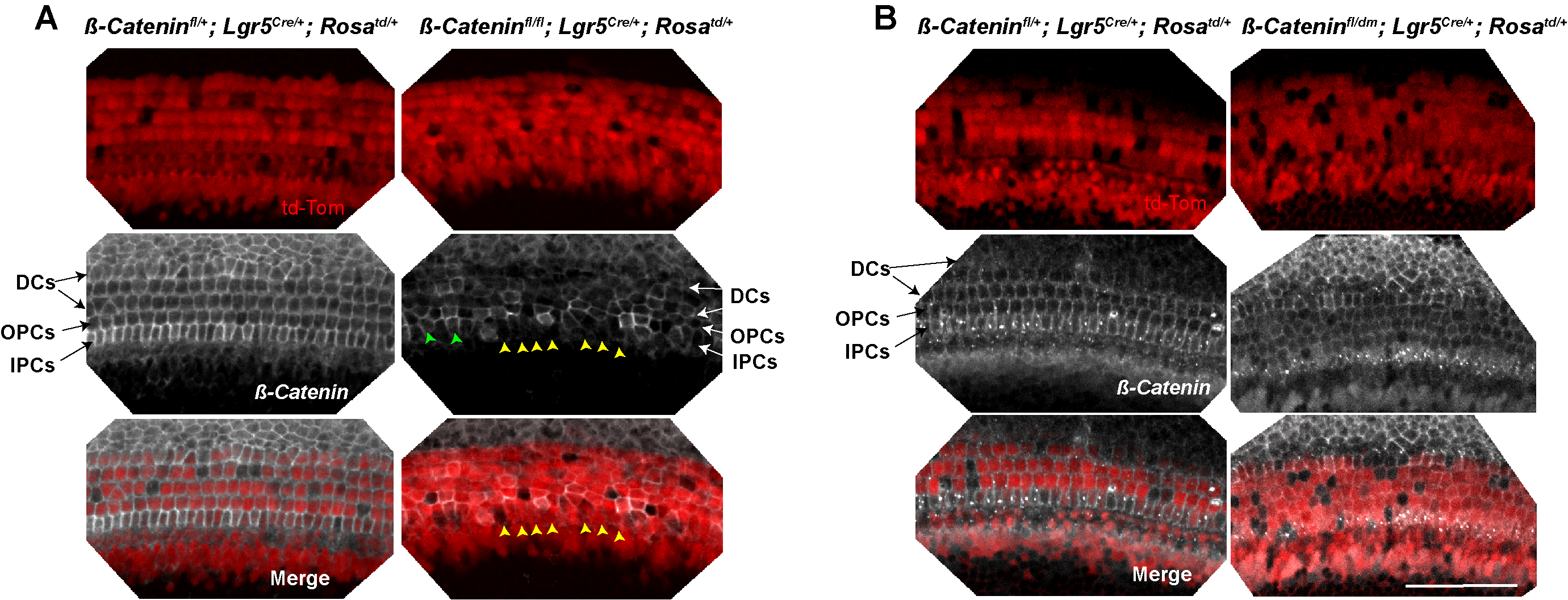

Supplement: S2 Fig — (A) Immunostaining of the basal turn whole mount E16.5 cochlear epithelium from β-Cateninfl/fl; Lgr5CreERT2/+; RosatdTomato/+ cochlea (full deletion) compared to control 48 hours post induction with tamoxifen (E14.5), showing staining for β-Catenin (white) along with tdTomato fluorescence (red) in cells with Lgr5CreERT2 recombination. The majority of IPCs along with other supporting cells shows loss of β-Catenin staining (yellow arrow heads), but a few supporting cells still show some β-Catenin expression (green arrow heads). (B) Immunostaining from β-Cateninfl/dm; Lgr5CreERT2/+; RosatdTomato/+ cochlea (transcriptional deletion) showing β-Catenin expression that is similar to controls. Scale bar = 100μm. (TIF) [file pgen.1010925.s002.tif]

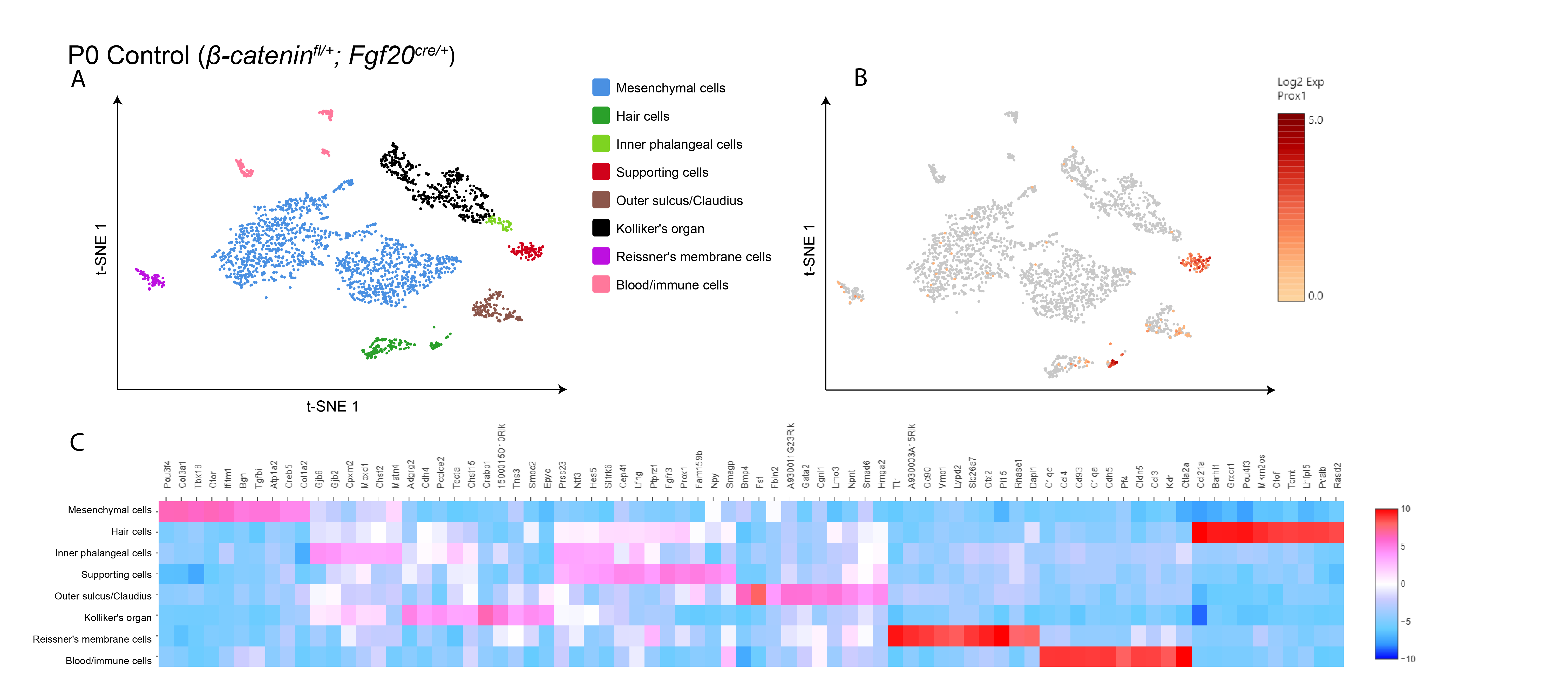

Supplement: S3 Fig — (A) t-SNE plot representing graph-based clustering of cochlear cells from P0 control (β-Cateninfl/+; Fgf20Cre/+) showing 8 different cell clusters (color-coded) identified based on known markers per population. (B) t-SNE plots representing Prox1+ expression levels within the supporting cell population in P0 control cochlear cells. (C) Differentially expressed genes (DEGs) within each identified cluster from (A) showing level of enrichment of each gene within each cluster (Loupe Browser v6.0, 10x Genomics). (TIF) [file pgen.1010925.s003.tif]

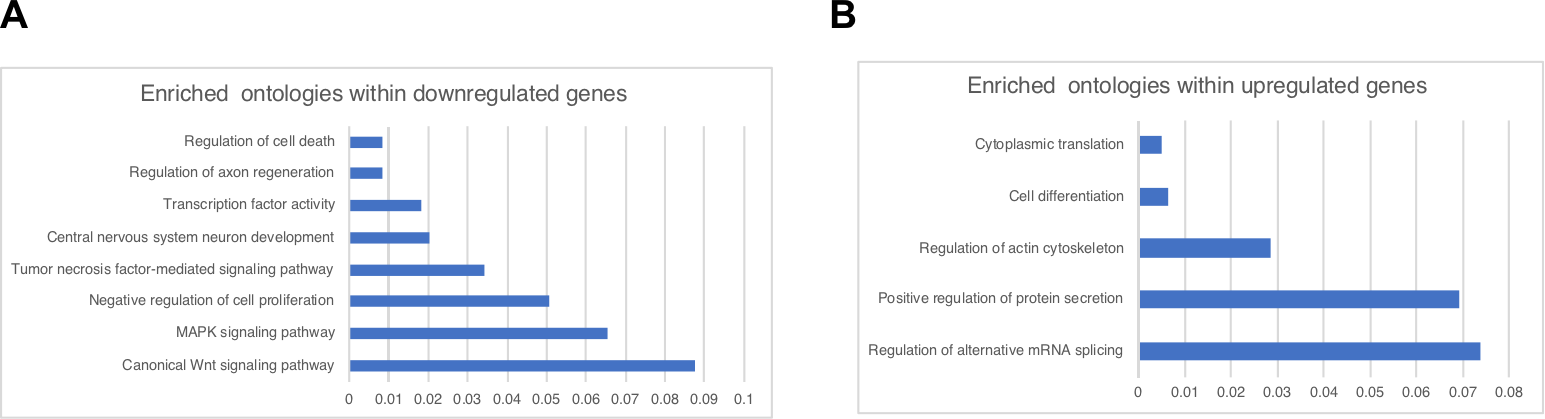

Supplement: S4 Fig — (A-B) Graphs representing enriched gene ontologies within differentially expressed genes in dm-cKO versus control Prox1+ cells (P values are shown on the x-axis). (TIF) [file pgen.1010925.s004.tif]

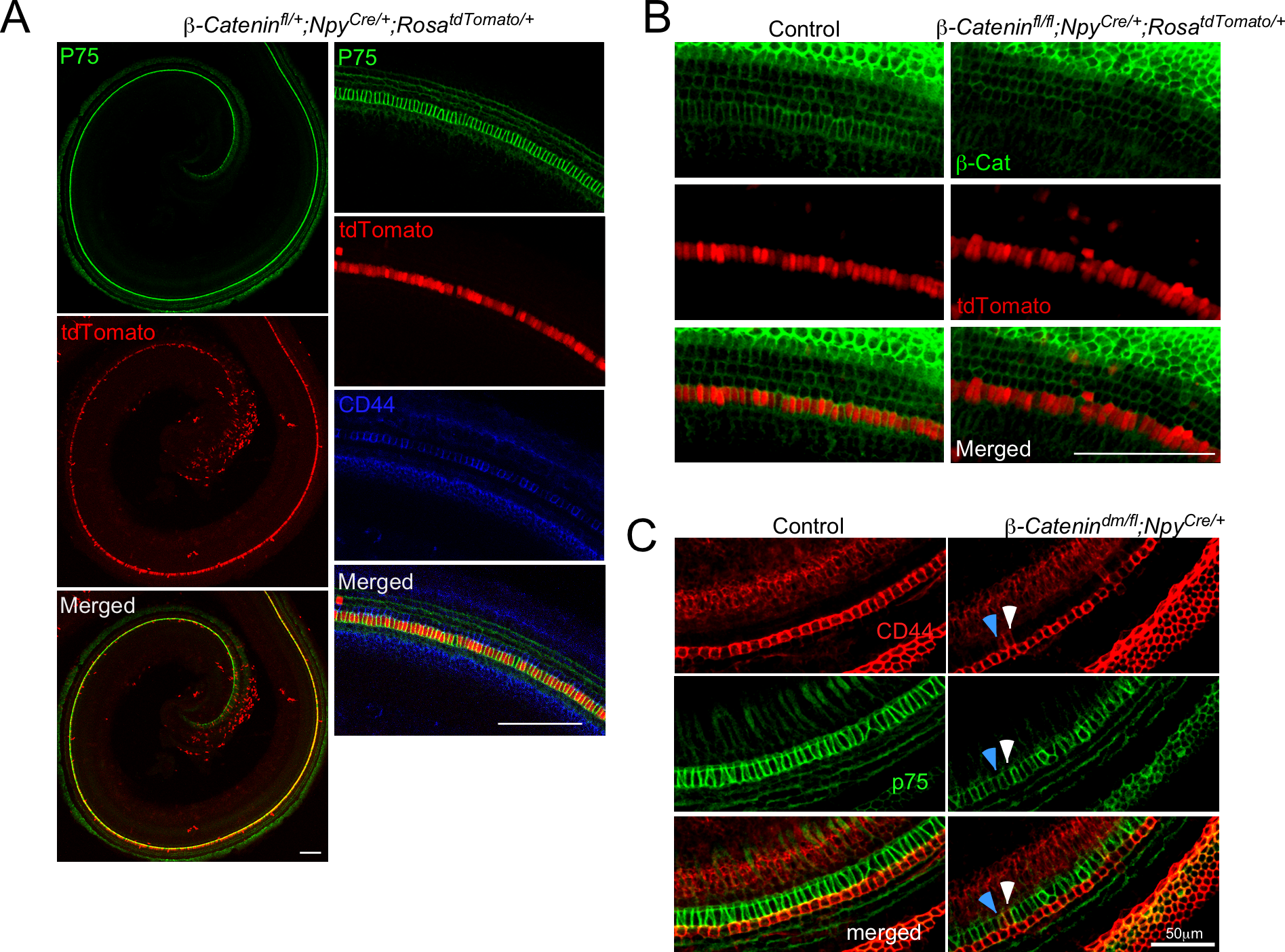

Supplement: S5 Fig — (A) Immunostaining of whole mount P0 cochlear epithelium from β-Cateninfl/+; NpyCre/+; RosatdTomato/+ cochlea showing p75 (green), tdTomato (red) and CD44 (blue) showing restricted Cre activity within IPCs. (B) Immunostaining of whole mount P1 cochlear epithelium from IPC-specific complete β-Catenin deletion (β-Cateninfl/fl; NpyCre/+; RosatdTomato/+) cochlea showing β-Catenin (green), tdTomato (red) showing efficient β-Catenin deletion within the IPCs. (C) Immunostaining of whole mount P1 cochlear epithelium from IPC-specific transcriptional β-Catenin deletion (β-Cateninfl/dm; NpyCre/+; RosatdTomato/+) cochlea showing p75 (green) and CD44 (red) showing examples for p75/Cd44 double-positive cells in IPC region (blue arrowhead) and Cd44+ ectopic cells (white arrowhead). Scale bar = 100μm unless otherwise specified. (TIF) [file pgen.1010925.s005.tif]
